# Supplementary material for: A generalizable and targeted molecular biopsy approach for in situ cryogenic electron tomography of vitreous brain tissue
Source: Cell Rep Methods. 2025 Jun 16;5(7):101080. doi: 10.1016/j.crmeth.2025.101080 (PMC12296509; doi:10.1016/j.crmeth.2025.101080)
Supplement: Document S1. Figures S1–S7 and Tables S1–S3 [file mmc1.pdf]

Cell Reports Methods, Volume *5*

Supplemental information

A generalizable and targeted molecular biopsy approach for *in situ* cryogenic electron tomography of vitreous brain tissue

Calina Glynn, Jake L.R. Smith, Matthew Case, Rebecca Csöndör, Ana Katsini, Maria E. Sanita, Thomas S. Glen, Avery Pennington, and Michael Grange

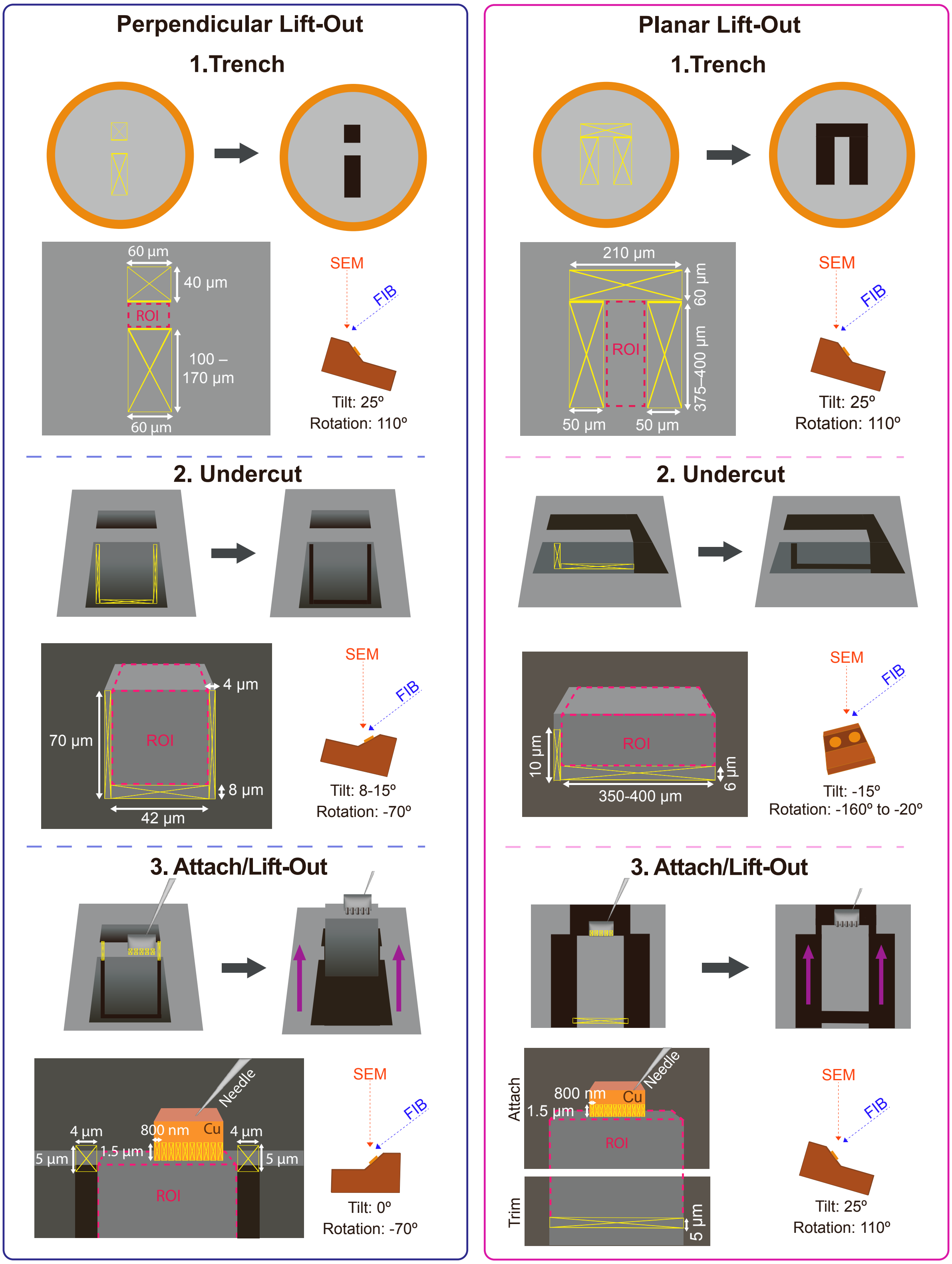

Perpendicular Lift-Out
1.Trench
60 µm
40 µm
ROI
100 – 170 µm
60 µm
SEM
FIB
Tilt: 25º
Rotation: 110º
2. Undercut
4 µm
70 µm
ROI
8 µm
42 µm
SEM
FIB
Tilt: 8-15º
Rotation: -70º
3. Attach/Lift-Out
Needle
4 µm
800 nm
Cu
4 µm
5 µm
1.5 µm
5 µm
ROI
SEM
FIB
Tilt: 0º
Rotation: -70º
Planar Lift-Out
1.Trench
210 µm
60 µm
ROI
375–400 µm
50 µm
50 µm
SEM
FIB
Tilt: 25º
Rotation: 110º
2. Undercut
ROI
10 µm
6 µm
350-400 µm
SEM
FIB
Tilt: -15º
Rotation: -160º to -20º
3. Attach/Lift-Out
Attach
800 nm
Needle
Cu
1.5 µm
ROI
Trim
5 µm
SEM
FIB
Tilt: 25º
Rotation: 110º

Figure S1. Milling Procedure for Cryo-Lift-Out from HPF Carriers. Related to Figure 1

Schematics depicting the workflow for perpendicular (left column, blue box) and planar (right column, pink box) lift-outs. Top – 1. Trench – diagrams showing the region of interest ('ROI', pink box), FIB milling patterns (yellow box) with measurements and stage orientations for trench milling in both perpendicular and planar samples. Middle – 2. Undercut – diagrams with FIB milling patterns (yellow box) to isolate the sample for lift out from the rest of the material for perpendicular and planar lift out. Note the difference in stage geometries for the planar lift out. Bottom – 3. Attach/Lift Out – diagram showing the redeposition FIB welding (yellow box) on the copper block (Cu) attached to the lift out needle. The purple arrow denotes the direction of lift out. Note the 180º difference in stage rotation for perpendicular versus planar lift out.

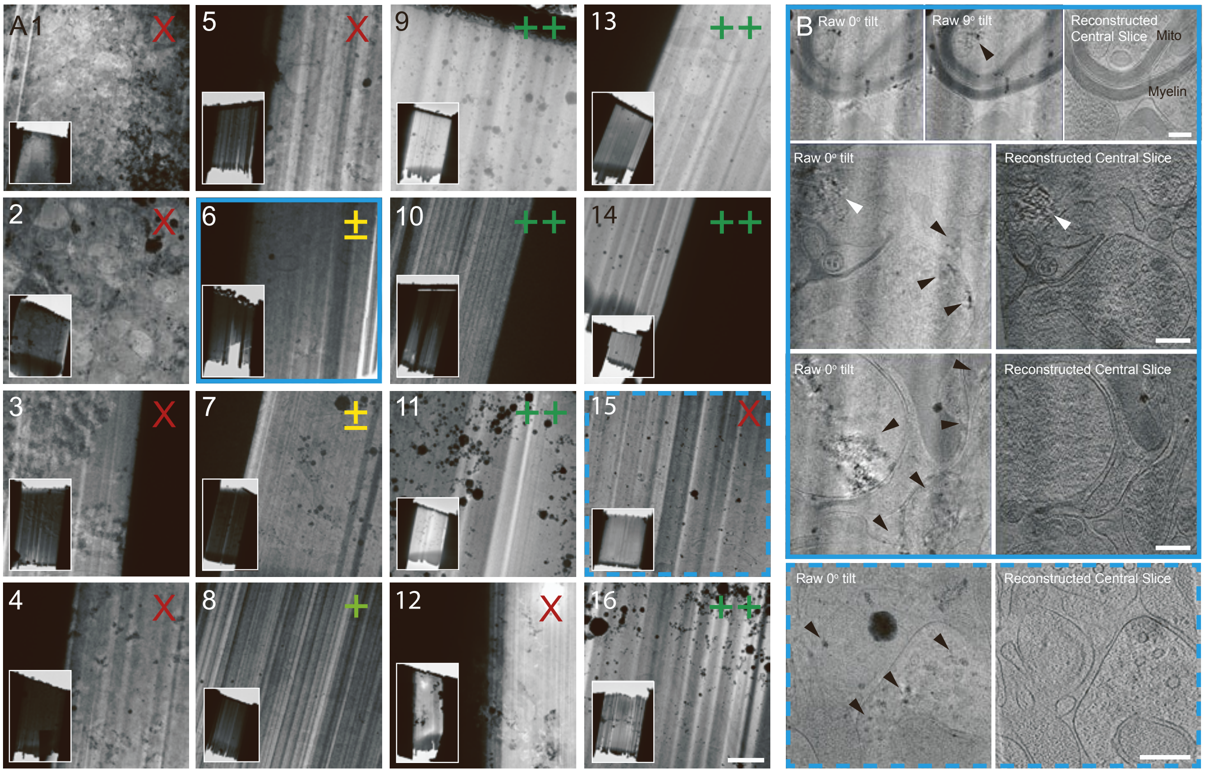

Figure S2. Vitrification Screen for Acute Mouse Brain Tissue Slices. Related to Figure 2.

(A) TEM of representative whole lamella overviews (left) and higher magnification search maps (right) from each tissue slice thickness, cryoprotectant, buffer, and incubation time combination detailed in Extended Data Table 1. ”X”, ”±”, ”+” and ”++” indicate degree of vitrification. An ”X” represents conditions where no areas of the lamella appeared vitreous and tilt series were not able to be collected without stage tracking errors resulting from extreme fluctuations in contrast introduced by ice diffraction. A ”±” indicates lamella where tilt series could be acquired, but frames in all tilt series displayed incomplete vitrification evidenced by ice diffraction. A ”+” rating was given to conditions where some tilt series could be acquired without ice diffraction, but some non-vitreous ice could be seen in some tilt series. A ”++” symbol was assigned to conditions where no or very few (<10%) tilt series displayed evidence of non-vitreous ice. Scale bar 2 μm.

(B) Representative raw tilts from condition 6 (top, solid outline) and condition 15 (bottom, dashed outline) illustrating that while signs of non-vitreous ice may (white arrows) or may not (black arrows) be present in tomogram reconstructions, raw tilts often displayed signed of non-vitreous ice. Scale bars: 200 nm.

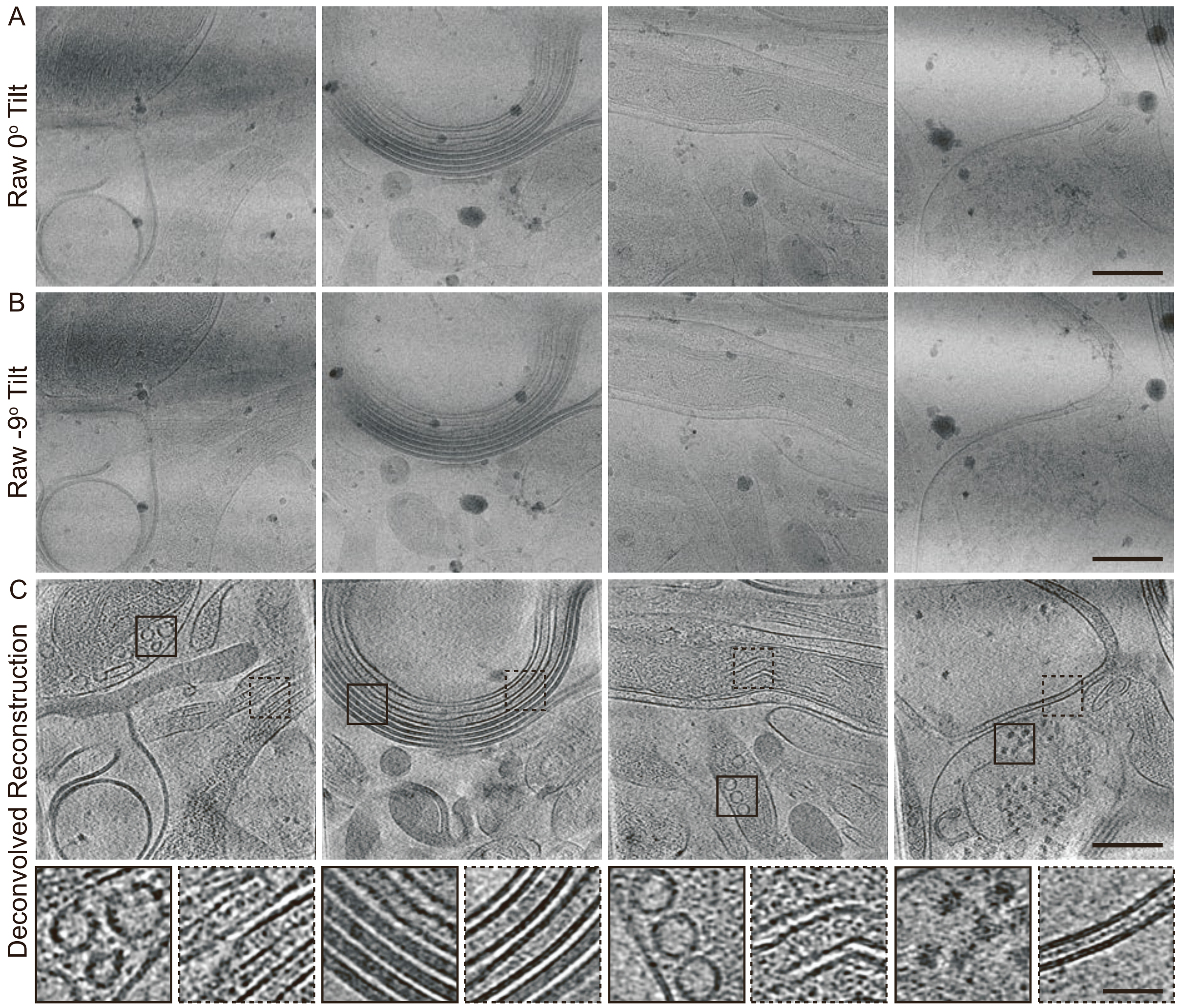

Figure S3. CryoET of Vitrified Mouse Cortex. Related to Figure 2.

(A–B) Raw tilts taken at 0 and –9º relative to the milling angle respectively, showing no diffraction from non-vitreous ice or membrane deformation indicative on a non-vitreous sample. Scale bar 200 nm

(C) Deconvolved tomogram reconstructions highlighting vesicles, microtubules, myelin, mitochondrial cristae, and ribosomes. Scale Bar in full image 200 nm, inset 50 nm

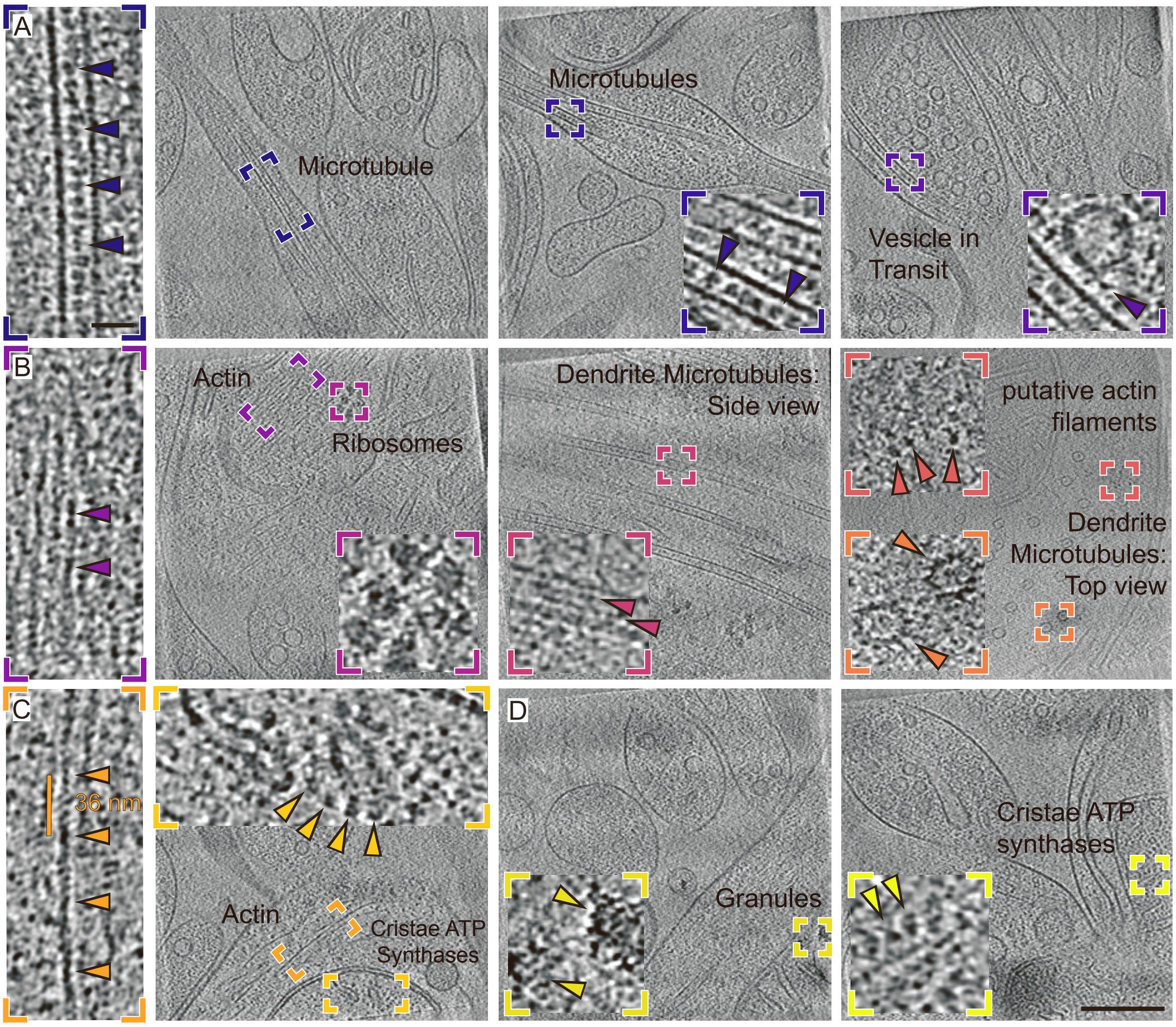

Figure S4. Features Observed in CA1-sr. Related to Figure 3.

(A) In perpendicular lift-outs from the CA1-sr we were able find abundant microtubules with luminal bodies (left and middle arrows) and in rare instances vesicles being transported along microtubules (right).

(B) We also less commonly found larger cytoskeletal organisation in the form of actin filaments (left), dendrites with microtubules traversing the entire field of view (middle, 10/359 tomograms) and very rarely head on views of dendrites with microtubule (right, bottom inset, 1/359 tomograms) and putative actin filament cross sections (right, top inset). For traversing microtubules, we could occasionally observe the lattice (arrows in middle inset) of aligned subunits.

(C) In our high-quality tomograms, actin could more clearly be identified by a 36 nm helical repeat (left inset). We were also able to independently identify cristae decorated with putative ATP synthases (top inset).

(D) We captured mitochondria with (left, 13/359 tomograms) and without (right, 119/359 tomograms) granular deposits. Putative ATPases could be observed in cristae (right inset, arrows). Scale bar for tomograms 200 nm, insets 25 nm.

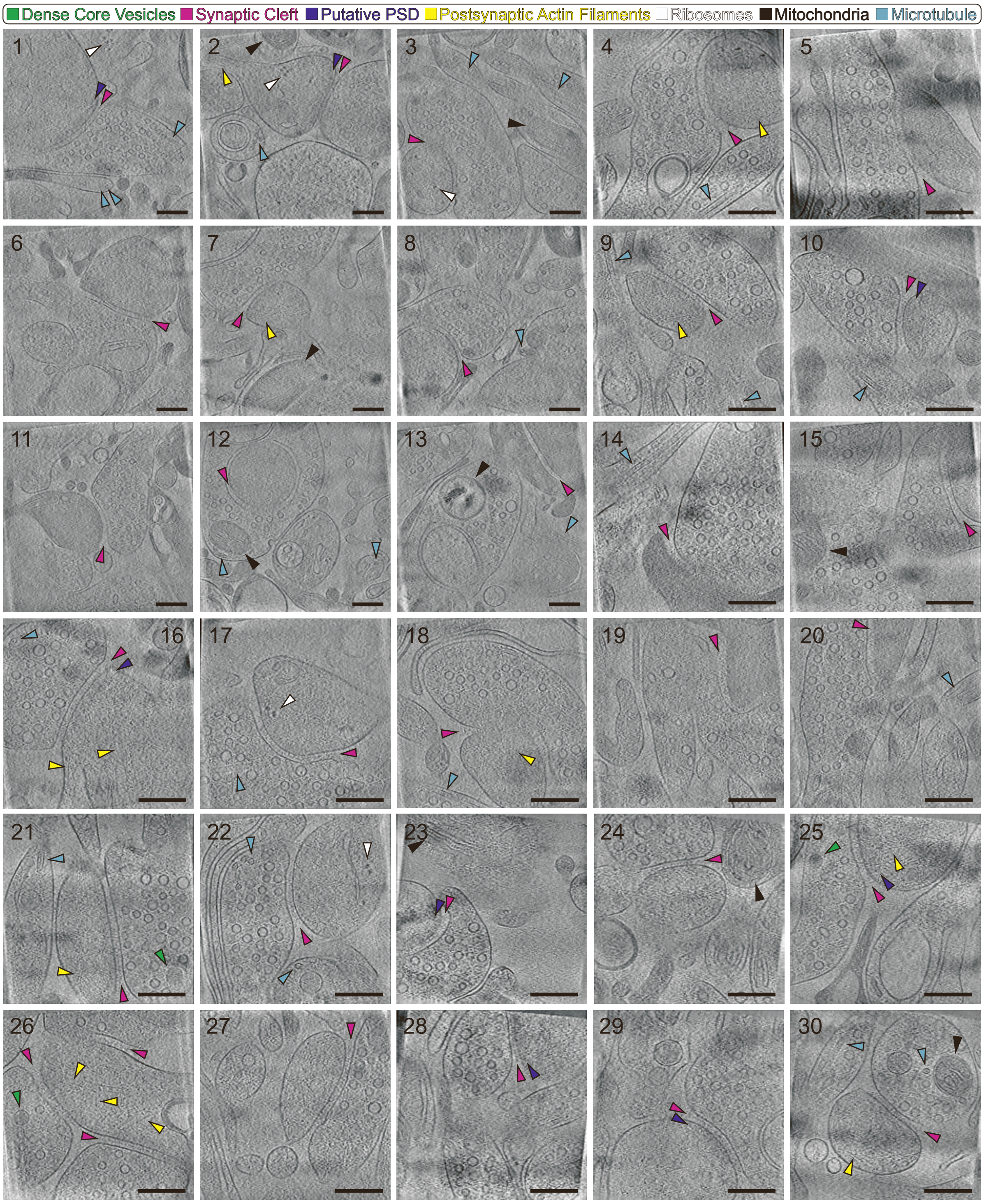

Figure S5. Synapse diversity in the CA1-sr. Related to Figures 4 and 5.

An array of 30 out of 107 total synapses representing all datasets including synapses from the planar lift-out (23–25, 28–30). Arrows highlight the synaptic cleft (pink) in all of the tomogram cross sections, dense core vesicles (blue) in the presynaptic terminal, putative postsynaptic density (PSD, dark purple) when present, postsynaptic actin filaments (light purple), ribosomes (white), mitochondria (orange) and microtubules (teal). Scale bars 200 nm.

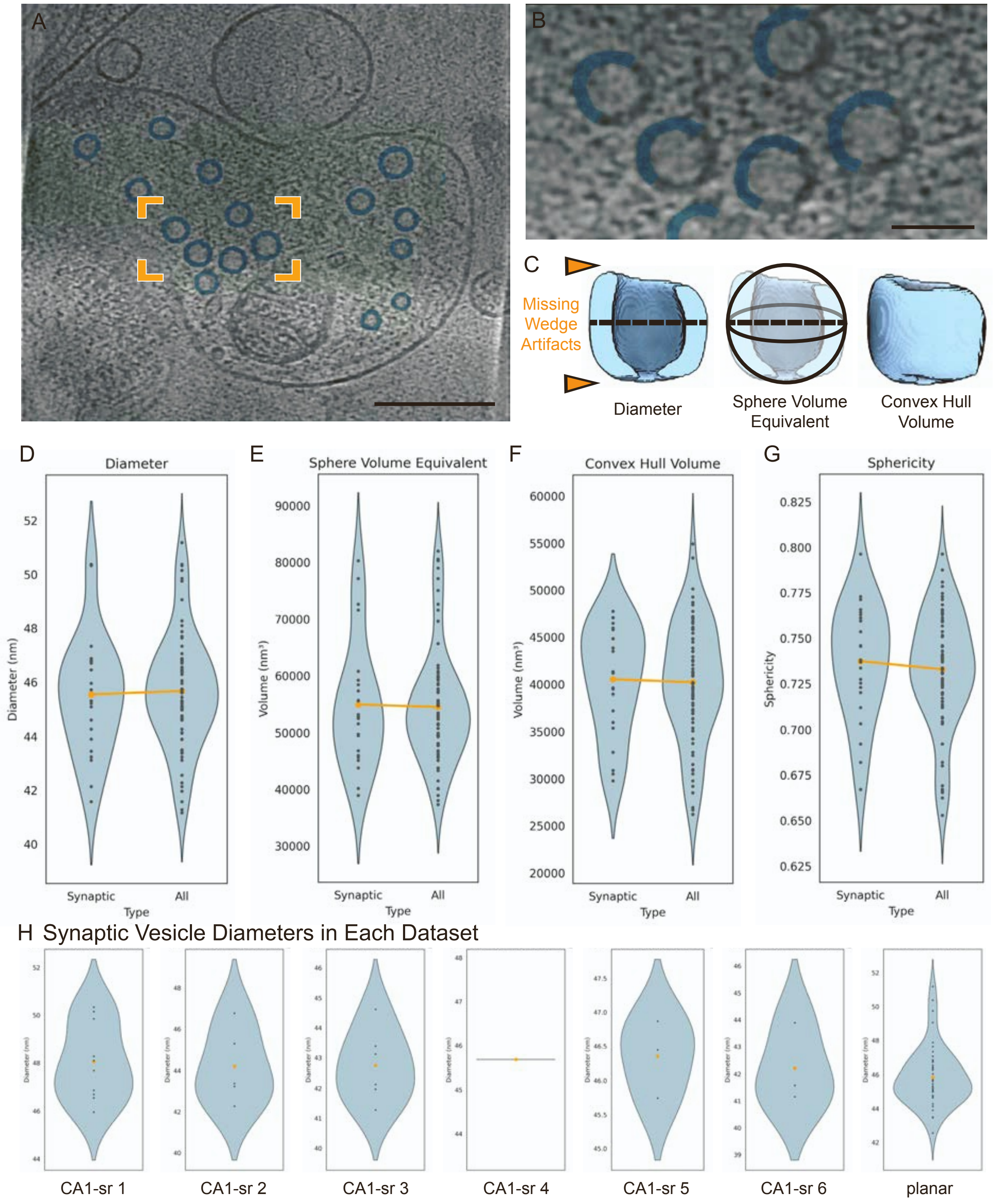

A
B
C
Missing Wedge Artifacts
Diameter
Sphere Volume Equivalent
Convex Hull Volume
D
Diameter
Diameter (nm)
Synaptic
All
Type
E
Sphere Volume Equivalent
Volume (nm³)
Synaptic
All
Type
F
Convex Hull Volume
Volume (nm³)
Synaptic
All
Type
G
Sphericity
Sphericity
Synaptic
All
Type
H Synaptic Vesicle Diameters in Each Dataset
CA1-sr 1
CA1-sr 2
CA1-sr 3
CA1-sr 4
CA1-sr 5
CA1-sr 6
planar

Figure S6. Segmentation and Size Distribution of Synaptic Vesicles. Related to Figures 4 and 5.

(A) The image shows a tomogram with 16 visible segmentations (62 vesicles in total throughout the tomogram). Scale bar: 200 nm.

(B) A crop of the tomogram with 6 vesicles, where half of the vesicle has been removed to show the degree to which the segmentation aligns with the vesicle boundaries. Scale bar: 50 nm.

(C) Illustration of the measurements used in the subsequent graphs, where diameter measures the widest diameter of the vesicle in 2D, sphere volume equivalent uses this diameter to calculate the vesicle volume assuming the vesicle is a perfect sphere, and the convex hull volume based on the volume that could encompass all points in the segmentation. All measures include the lipid bilayer.

(D) Violin plot of the mean diameter, with left plot (Synaptic) including only tomograms where all vesicles are in a visible synapse (27) and the right plot (All) includes all vesicles in all synapse tomograms (107) which could be segmented. The diameter is measured in 2D, per slice, where dots are the per-tomogram means of the median of the per-slice 2D feret diameter of each vesicle. The median was chosen to be more robust to outliers. There was no statistically significant difference between the diameters of vesicles that were part of a visible synapse and vesicles where an accompanying synapse was not captured within the tomogram.

(E) Plot of the mean Sphere Volume Equivalent per tomogram, which is the calculated volume of a sphere, given the diameter used in (D). As in (D), there was no statistically significant difference in volume between the two groups.

(F) The mean volume of the convex hull of the segmented connected component, per tomogram. The segmented component is a is the shell of the vesicle, so the convex hull is used to fill in the interior. There was no statistically significant difference found between the two groups.

(G) A violin plot of the mean sphericity of the vesicles, per tomogram. There was no statistically significant difference found between the two groups.

(H) Synaptic vesicle diameter distribution for each dataset. There was no statistically significant difference found in synaptic vesicle diameter between any of the datasets.

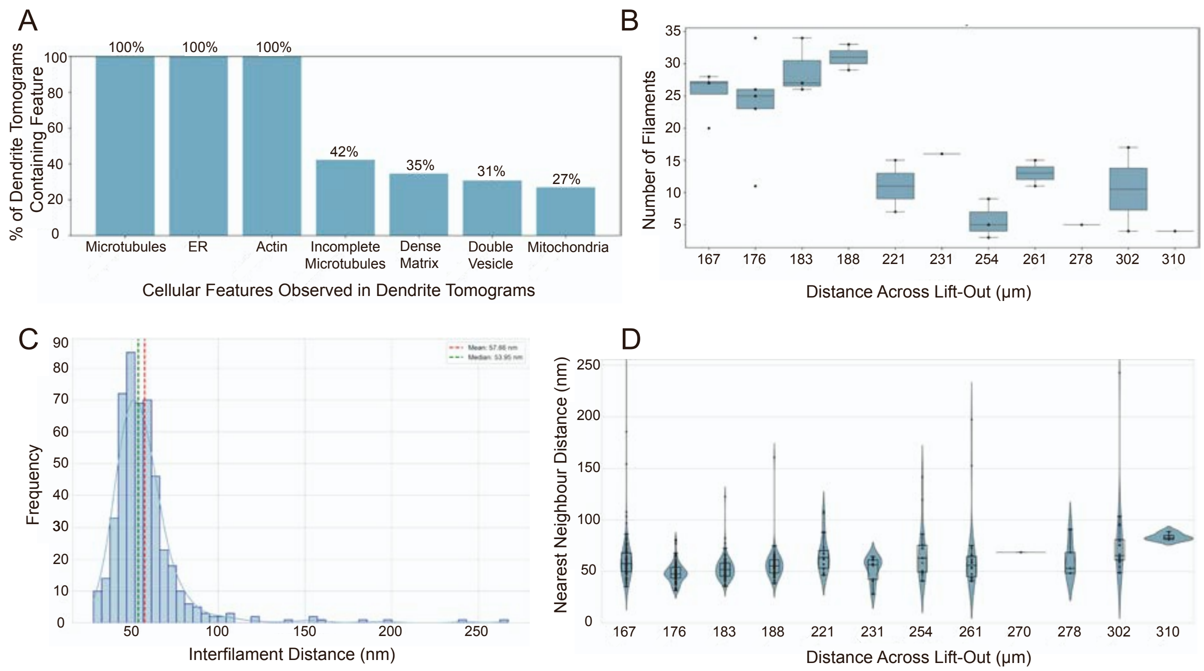

Figure S7. Analysis of the cytoskeletal composition and organization of the apical dendrite network. Related to Figures 6 and 7.

(A) Characterization of the percentage of the cellular components observed in 30 reconstructed tomograms depicting axial views of dendrites.

(B) The number of microtubules observed in dendrite tomograms as a function of distance from the CA1-so.

(C) Histogram of the nearest neighbour differences between microtubules from all 30 dendrite tomograms. Mean and median values are plotted as red and green dashed lines respectively.

(D) Violin plot of nearest neighbour distances between microtubules as a function of distance from the CA1-so.

Table S1. Conditions assessed for vitrification state of high-pressure frozen tissue. Related to Star★Methods sections: Experimental Model and Subject Details – Animal Handling and Brain Dissection and Method Details – High Pressure Freezing.

Mouse Age (days)	Brain Region	Section Thickness (µm)	Carrier Material	Carrier Recess Depth (µm)	Cryoprotectant and Buffer	Incubation Time (min)	Vitreous Rating[$]	Figure S2 Number
14	Cortex	200	Aluminium	200	20% Dextran+ in PBS pH 7.4	0	X	1
13	Cortex	150	Aluminium	150	20% Dextran in PBS pH 7.4	30	X	2
7	Cortex	150	Copper	200	20% Dextran in PBS pH 7.4	30	X	3
118	Cortex	150	Copper	200	20% BSA in 100 mM PB pH 7.4	35	X	4
118	Cortex	100	Copper	100	20% BSA in 100 mM PB pH 7.4	0	X	5
9	Cortex	150	Copper	200	10% Dextran 5% Sucrose in 100 mM PB pH 7.4	25	X	
38	Cortex	100	Copper	100	10% Dextran 5% Sucrose in 100 mM PB pH 7.4	20	+*	
125	Cortex	100	Copper	100	10% Dextran 5% Sucrose in 100 mM PB pH 7.4	25	±	6
125	Cortex	100	Copper	100	10% Dextran 5% Sucrose in 100 mM PB pH 7.4	30	±	7
125	Cortex	100	Copper	100	10% Dextran 5% Sucrose 5% Ethylene Glycol in 100 mM PB pH 7.4	20	+	8
149	Cortex	100	Copper	100	10% Dextran 5% Sucrose 5% Ethylene Glycol in 100 mM PB pH 7.4	25	++	9
125	Cortex	100	Copper	100	10% Dextran 10% Sucrose in 100 mM PB pH 7.4	20	++	10
149	Cortex	100	Copper	100	10% Dextran 10% Sucrose in 100 mM PB pH 7.4	25	++*	11
184	Hippocampus	100	Copper	100	10% Dextran 10% Sucrose in NMDG pH 7.4	0	X	12
184	Hippocampus	100	Copper	100	10% Dextran 10% Sucrose in NMDG pH 7.4	15	++	13
184	Hippocampus	100	Copper	100	10% Dextran 5% Sucrose 5% Ethylene Glycol in aCSF[a] pH 7.4	20	++	14
144	Cortex	100	Copper	100	20% Dextran in NMDG pH 7.4	25	X	15
144	Cortex	200	Copper	200	10% Dextran 10% Sucrose in NMDG pH 7.4	30	++	16

[$]For vitreous rating, "X", "±", "+" and "++" indicate degree of vitrification. An "X" represents conditions where no areas of the lamella appeared vitreous and tilt series were not able to be collected without stage tracking errors resulting from extreme fluctuations in contrast introduced by ice diffraction. A "±" indicates lamella where tilt series could be acquired, but frames in all tilt series displayed incomplete vitrification evidenced by ice diffraction. A "+" rating was given to conditions where some tilt series could be acquired without ice diffraction, but some non-vitreous ice could be seen in some tilt series. A "++" symbol was assigned to conditions where no or very few (<10%) tilt series displayed evidence of non-vitreous ice.

[+]Dextran 40,000 MW
*Conclusion based on one tilt series
[a]aCSF composition 119 mM NaCl, 26.2 mM NaHCO3, 2.5 mM KCl, 1 mM Na2HPO4, 1.3 mM MgCl2, 10 mM glucose, 2.5 mM CaCl2

Table S2. Trench milling parameters for perpendicular and planar cryo-lift-outs from HPF carriers. Related to Star★Methods – Method Details – Cryo-Lift-Out.

Step	Sub-step	Current	Pattern Type	Pattern Dimensions (µm)			Time (min)	Notes
				X	Y	Z		
Fluorescence Overview	NA	NA	NA	NA	NA	NA	20-50	Time for imaging with 1 fluorophore, 20 minutes for 3 Z steps, 50 minutes for 12 Z steps.
Fiducials	NA	4-60 nA	Rectangle	75	4	3	10	Used for alignment between SEM and IFM.
Trenches: Perpendicular	Long Trench	60 nA	RCS	60	150-200	5-6	<30	For a desired lift-out length of ~60-70 um. Longest long trenches should be used in conjunction with deepest deep trenches. For 100 um thick samples, long trench lengths <170 um and deep trench depths <9 should be used to avoid milling into the metal carrier. Largest lift-outs yield ~10-14 3-5 µm thick sections. RCS patterns mill towards target region.
	Deep Trench	60 nA	RCS	60	40-60	6-11		
Trenches: Planar	Side Trenches	200 nA	RCS	50	350-400	6	7–8	For a desired lift-out length of ~350-400 µm. Trenches are milled symmetrically at either side of the region of interest, with the scan pattern directed towards the area for lift out.
	Top Trench	60 nA	RCS	210	50	2	6	To allow the lift-out needle with the copper block attachment to be brought to the top of the sample. The scan pattern is directed towards the area for lift out.
Side and Undercuts: Perpendicular	Side Cuts	15 nA	Rectangle	4	40-60	4	10	Use a stage tilt of <13° to avoid milling into the metal carrier for deep lift-outs.
	Undercut	15 nA	Rectangle	53-58	6-8	4		
Side and Undercuts: Planar	Undercut	15 nA	Rectangle	350-400	6	1	16–20	Perform undercuts from both ±90° stage rotation relative to the region of interest. The time listed is for milling from one of the angles.
Weld Copper Block to Tissue	NA	0.3 nA	CCS	1	2	3	< 5	Tilt the stage to the shallowest angle where remaining material from side cuts is still visible, roughly -3 to 3° . Milling patterns should be oriented to mill from tissue towards the copper block. After attachment, then mill away remaining side material. Dwell time was set to 30 µs
Sectioning and Welding	Sectioning	1-4 nA	Line	~50	NA	4	2-5	The stage was tilted to the shallowest angle possible, around –3 to -5°, for section deposition. 4 nA allows for faster cuts but 1 nA results in a smoother surface that is advantageous for subsequent thinning steps.
	Welding	0.3 nA	CCS	3	0.8	5	3–4 per section	Stage was tiled to 15° before placing welding patterns. Welds were placed with 4 um periodicity such that each section had 3-4 welds per side with 6-8 welds per section in total. CCS patterns milled from the section towards the copper bars. Dwell time was set to 30 µs and each welding pattern took 30 seconds to mill.

Table S3. Datasets generated from vitreous samples collected in this work. Related to Figures 2–7.

Mouse Sex	Mouse Age	Hemisphere (L/R)	Cryoprotectant and Buffer	Incubation Time (min)	Region	Dataset Number (for CA1-sr)	Tilt Series Collected	Synapses	% Dataset that is Synapses	Average Synaptic Vesicle Diameter (nm)
M	184	Not recorded	10% Dextran, 10% Sucrose in NMDG, pH 7.4	15	CA1-sr	1	58	16	28	48.0
M	184	L	10% Dextran, 5% Sucrose, 5% Ethylene Glycol in aCSF, pH 7.4	20	CA1-sr	6	10	3	30	42.2
M	184	L	10% Dextran, 10% Sucrose in aCSF, pH 7.4	20	CA1-sr	5	22	5	23	46.4
F	172	R	10% Dextran, 10% sucrose in NMDG, pH 7.4	20	CA1-sr	2	145	19	13	44.1
M	184	R	10% Dextran, 10% sucrose in NMDG, pH 7.4	15	CA1-sr	3	60	11	18	42.7
F	172	R	10% Dextran, 10% sucrose in NMDG, pH 7.4	20	CA1-sp		35	0	0	N/A
F	172	R	10% Dextran, 10% sucrose in NMDG, pH 7.4	20	CA1-sr	4	64	2	3	45.7
M	184	L	10% Dextran, 10% sucrose in NMDG, pH 7.4	15	CA1*		252	51	20	45.9
F	144	Not recorded	10% Dextran, 10% sucrose in NMDG, pH 7.4	30	cortex		28	0	0	N/A

* CA1 was sampled from CA1-so to CA1-slm
