# Supplementary material for: A generalizable and targeted molecular biopsy approach for in situ cryogenic electron tomography of vitreous brain tissue
Source: Cell Rep Methods. 2025 Jun 16;5(7):101080. doi: 10.1016/j.crmeth.2025.101080 (PMC12296509; doi:10.1016/j.crmeth.2025.101080)
Supplement: Document S2. Article plus supplemental information [file mmc2.pdf]

Cell Reports
Methods

Article

A generalizable and targeted molecular biopsy approach for *in situ* cryogenic electron tomography of vitreous brain tissue

Graphical abstract

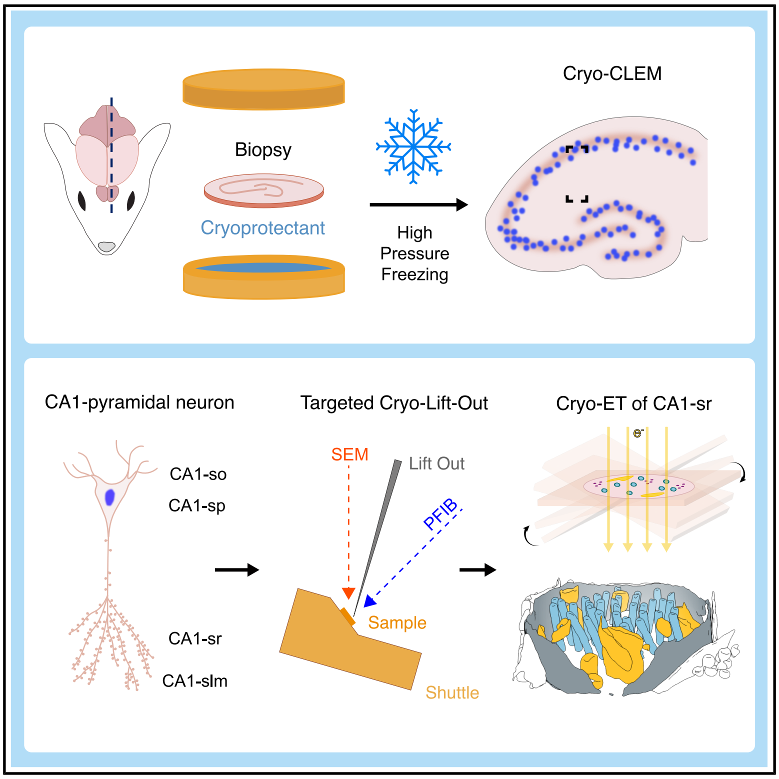

Authors

Calina Glynn, Jake L.R. Smith, Matthew Case, ..., Thomas S. Glen, Avery Pennington, Michael Grange

Correspondence

michael.grange@rfi.ac.uk

In brief

Glynn et al. present a workflow for routine analysis of mammalian brain tissue by cryogenic electron tomography. The combination of robust sample vitrification, correlated cryo-fluorescence microscopy, and plasma focused ion beam milling is utilized to demonstrate a high-throughput and targeted approach for structural investigations in mouse hippocampal tissue.

Highlights

- Robust vitrification of brain tissue enables structural biology in tissues
- Fluorescence maps of whole cryogenic biopsies allow specific sublayer targeting
- Sub-nanometer visualization of synapses and dendritic cytoskeleton using cryo-ET
- Flexible lift-out geometries unlock alternative views of neuronal organization

Glynn et al., 2025, Cell Reports Methods *5*, 101080
July 21, 2025 © 2025 The Authors. Published by Elsevier Inc.
https://doi.org/10.1016/j.crmeth.2025.101080

CellPress

Cell Reports Methods

CellPress OPEN ACCESS

Article

A generalizable and targeted molecular biopsy approach for *in situ* cryogenic electron tomography of vitreous brain tissue

Calina Glynn,[1,4] Jake L.R. Smith,[1,2,4] Matthew Case,[1] Rebecca Csöndör,[1,3] Ana Katsini,[1] Maria E. Sanita,[1] Thomas S. Glen,[1] Avery Pennington,[1] and Michael Grange[1,2,5,*]
[1]The Rosalind Franklin Institute, Harwell Science & Innovation Campus, Didcot OX11 0QS, UK
[2]Division of Structural Biology, Wellcome Centre for Human Genetics, University of Oxford, Oxford OX3 7BN, UK
[3]Queen's Square Institute of Neurology, University College London, London WC1N 3BG, UK
[4]These authors contributed equally
[5]Lead contact
*Correspondence: michael.grange@rfi.ac.uk
https://doi.org/10.1016/j.crmeth.2025.101080

MOTIVATION Studying the 3D structure and organization of cells and proteins is essential for understanding cellular function. Current state-of-the art approaches visualize proteins within cells grown in dissociated cultures, which does not sufficiently recapitulate the cellular environment *in vivo*. To perform these experiments within a more biologically relevant model (i.e., a tissue), significant technical challenges must be overcome. Here, we demonstrate how we can routinely preserve tissue samples by vitrification and produce thin sections amenable to structural biology using plasma focused ion beam milling, cryo-lift-out, and cryogenic electron tomography. In combination with cryo-light microscopy, we can generate structural datasets of sub-regions within mammalian hippocampus at scale. This approach is generalizable and can be extended to facilitate routine structural investigation in other tissues.

SUMMARY

Cellular cryogenic electron tomography (cryo-ET) enables the capture of detailed structural information within a biologically relevant environment. However, information in more complex samples, such as multicellular specimens and tissues, is lacking. Importantly, these observations need to be set in the context of populations. Currently, imaging on the molecular scale is limited to a few observations *in situ* that struggle to be generalized. This is due to limitations in throughput and versatility employed by current instrumentation. Here, we utilize plasma focused ion beam milling to examine the molecular landscape of mouse hippocampus by cryo-ET. We reveal the complex organization of macromolecules in targeted regions across CA1 stratum pyramidale (sp) to radiatum (sr), representing a molecular atlas of hippocampal architecture in adult mice. The combination of instrumentation and application of technical advancements provides a framework to explore specific structural questions within other tissues in a targeted manner.

INTRODUCTION

Within the brain, a myriad of molecular interactions are responsible for cellular connectivity, homeostasis, and maintenance. Characterizing the organization of molecules within the context of brain tissue could unlock fundamental mechanistic insight into a range of processes, with consequences for our understanding of health and disease. This challenge becomes increasingly difficult with scale, where multiple subjects are needed to understand disease-related genotype-phenotype relationships. High-throughput imaging tools that bridge the gap between cellular structure and (dys)function are needed.

Cryogenic electron microscopy (cryo-EM) is a powerful tool used to observe the structure of proteins that can be harnessed directly inside cells by cryogenic electron tomography (cryo-ET). For cryo-ET, samples must be preserved in a frozen, hydrated state before molecules can be visualized to sub-nanometer resolution by sub-volume averaging.[1–3] Sample preservation by vitrification for cellular samples is typically achieved through plunge freezing into liquid ethane, whereas tissue biopsies, which are typically >100 μm in thickness (still >10-fold thicker than cells), require high-pressure freezing (HPF). Consistent and robust sample preservation currently limits routine structural analysis in tissues by cryo-ET.

© 2025 The Authors. Published by Elsevier Inc.
This is an open access article under the CC BY license (http://creativecommons.org/licenses/by/4.0/).

To image molecules within cells using cryo-ET, the sample must be sufficiently thin to allow the transmission of electrons. Creating electron-transparent lamellae of ∼100–200 nm thickness from >100 μm thick vitrified tissues presents a technical challenge for cryo-ET investigations. Cryo-EM of vitreous sections (CEMOVIS), or ultramicrotomy, can be used to section ribbons of tissue down to 40 nm in thickness[4] but suffers from multiple artifacts,[5–8] which can obfuscate detail. Cryo-focused ion beam milling coupled with scanning electron microscopy (FIB/SEM) is now routinely used to create thin lamellae in cellular samples.[9–12] This produces fewer artifacts compared to sections created using CEMOVIS and is compatible with structure determination in cells.

Traditional liquid metal ion source (LMIS) FIB/SEMs have been demonstrated to work with samples of ∼50 μm depth.[13,14] Alternatively, plasma FIBs (PFIBs) have recently been utilized for cryogenic life science samples,[15] both for volume EM[16] and lamella fabrication, allowing for high-resolution sub-volume averaging.[17–19] The increased sputter yield of xenon plasma allows for the excavation of large volumes in a shorter time frame,[17,20] opening up possibilities for exploring tissues with cryo-ET in a more high-throughput fashion.

Lamella fabrication from samples high-pressure frozen directly onto EM grids has been demonstrated.[14,21] This can result in the compression of material during HPF, deforming morphological features within the native tissue.[21] Alternatively, biopsies can be frozen in specialized HPF carriers with a thickness of up to 200 μm, allowing vitrification without grid-induced distortions. However, these carriers are not electron transparent, and removal of a targeted region is needed to enable subsequent cryo-ET. One such approach used to achieve this is cryo-lift-out. With a single cryo-lift-out, a series of spatially related lamellae across entire organisms and multicellular samples on grids can be made[13,22] though due to the use of LMIS FIB/SEMs, this is still limited to depths of samples 30–50 μm thick. By utilizing PFIB/SEM with its greater sputter yield, serial cryo-lift-out has the potential to be extended to >100 μm tissue samples in HPF carriers.

Here, we present our workflow to facilitate routine structural investigation in mammalian brain tissue. We demonstrate robust vitrification of mouse brain by HPF within 3 h postmortem. Utilizing cryo-fluorescence microscopy and PFIB milling, we adapt a cryo-lift-out strategy to image specific sub-regions of the CA1 region of hippocampus from samples frozen in HPF carriers up to 200 μm thick. This enabled features, particularly synapses and the apical dendrite network, to be characterized within this layer by cryo-ET. Using biopsies from several mice and by sampling multiple regions within the brain, we exemplify the potential to investigate targeted structural biological questions across cohorts. Different sample geometries were accessed using a planar lift-out, extending the versatility of our approach to generate serial sections spanning CA1 strata pyramidale (CA1-sp) through radiatum (CA1-sr). This highlights an alternate view of the apical dendrite network not accessible by cellular cryo-ET. Ultimately, this work presents the basis for a high-throughput investigation of tissue by cryo-ET that has the potential to define the molecular underpinnings of disease *in situ*, an approach that, to date, has been difficult to implement in a targeted, reproducible manner.

RESULTS

Extraction of brain tissue from HPF carriers

Mouse hemibrains (∼6 months of age) were sectioned to 100–200 μm in thickness using a vibratome and high-pressure frozen in 100 or 200 μm planchettes (Table S1). Subsequently, sub-regions of hippocampus could be targeted and correlated using micron-scale cryo-fluorescence microscopy to nanometer-scale imaging by cryo-ET (Figure 1).

Using an adapted cryo-lift-out approach,[22] we developed two complementary procedures, perpendicular and planar, to extract regions from different geometries (Figures 1A and S1). These procedures allowed HPF tissue to be removed using different orientations relative to the tissue slice.

For a desired perpendicular lift-out length of ∼60–70 μm, 10–14 sections could be deposited with the time taken for each step outlined in Table S2 and described in the STAR Methods (Figure 1D). For planar geometries, the trench milling and undercuts are larger due to the increased size and orientation, which can increase the amount of time needed to remove the tissue section (Table S2). For both approaches described in this work, 26 lift-outs were extracted, resulting in 186 serial sections being deposited. Of these, 170 were retained (91.4%) upon transfer between microscopes for lamella fabrication.

After deposition, serial sections were subsequently thinned to electron transparency using a combination of xenon plasma (to ∼400–600 nm) and manual thinning with argon plasma (Figure 1E). This hybrid approach was used because xenon has a milling rate 3–4 times greater than argon, reducing milling time for bulk removal of material before thinning with the finer argon beam.[17] The resulting lamellae reached thicknesses comparable to those obtained for cellular lamellae and had contrast transfer functions (CTF) that could be accurately fitted to sub-nanometer resolution (Figure 2).

Brain tissue vitrification

We assessed the vitrification of mouse brain slices under different conditions, such as tissue thickness, cryoprotectant, and incubation conditions (Figure S2; Table S1). The visibility of non-vitreous ice in transmission electron microscopy (TEM) overview images of lamellae, ice diffraction in TEM images, and distortion of membranes were used as markers of poor vitrification. We assessed 18 conditions in total, using cryo-lift-out to empirically determine whether those conditions consistently led to vitreous ice. Several conditions led to partially vitrified preparations, where ice reflections are observed in certain images (Figure S2). The most robust vitrification was achieved via incubation for at least 15 min with combinations of cryoprotectants of at least 10% low molecular weight (MW) (here, sucrose MW = 0.34 kDa or ethylene glycol MW = 0.062 kDa) and higher MW (e.g., dextran MW = 40 kDa) components. Cryoprotectants containing only large MW components such as dextran or BSA (MW = 66.5 kDa) were insufficient to yield vitrified tissue, with samples exhibiting either dehydration artifacts, such as distorted membranes, or containing reflections in some tilt images (Figure S2B). Tilt series for subsequent analysis were only acquired in lamellae where we did not observe any of these features.

CellPress
OPEN ACCESS

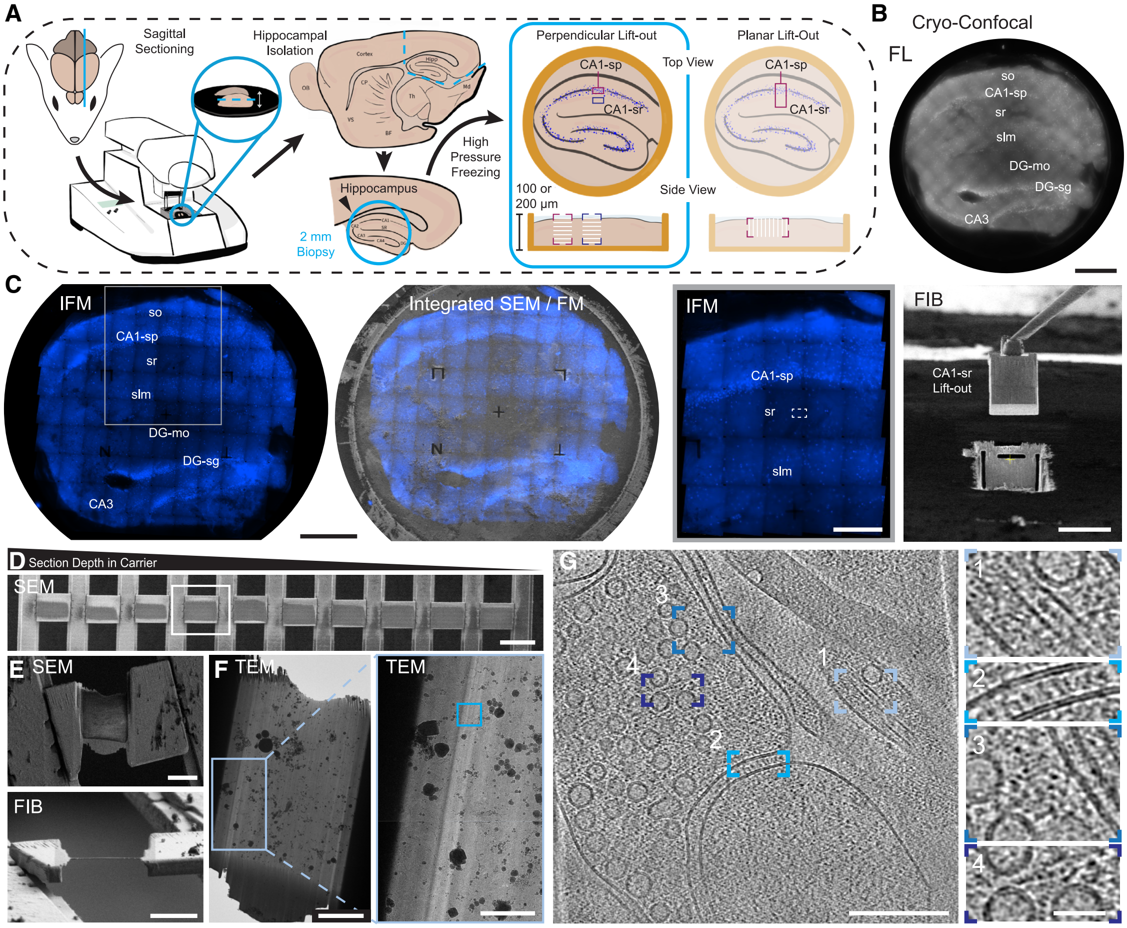

Figure 1. CLEM workflow for targeting features within vitrified mouse brain tissue

(A) Schematic of mouse brain sectioning and biopsy illustrating capacity to target specific layers within defined sections of mouse hippocampus for subsequent lift-out in perpendicular or planar geometries. In the rest of this figure, a perpendicular lift-out (boxed) was used.

(B) Cryo-confocal image of a vitrified mouse hippocampal section in a high-pressure freezing carrier. Sublayers for CA1 are abbreviated as stratum oriens (so), stratum pyramidal (sp), stratum radiatum (sr), and stratum lacunosum moleculare (slm), with sublayers for dentate gyrus abbreviated as dentate gyrus molecular layer (DG-mo) and dentate gyrus stratum granule (DG-sg). Scale bar: 500 μm.

(C) The same section imaged using the integrated fluorescence module (IFM) in the FIB/SEM (left, scale bar: 500 μm) with CA1-sr lift-out target and lift-out marked (right, IFM scale bar: 200 μm, FIB scale bar: 50 μm).

(D) Serial sections from CA1-sr lift-out with sections deeper in the carrier on the left and closer to the tissue surface at the right. Scale bar: 50 μm.

(E) Thinned section boxed in (D) from SEM (top) and FIB (bottom) views. Scale bars: 10 μm.

(F) Lamella of the same section in the TEM (scale bar: 5 μm) with boxed region enlarged (right, scale bar: 2 μm).

(G) Reconstructed tomogram from region boxed in the right image showing a synapse (scale bar: 200 nm) with details such as microtubules (box 1), densities in the synaptic cleft (box 2), and densities spanning between synaptic vesicles and membranes (boxes 3–4). Scale bar for insets: 40 nm.

To assess the quality of brain cryo-fixation, mouse cortex was vitrified and analyzed by cryo-ET. We were able to observe common cellular and brain-specific features, including myelin, synaptic vesicles, mitochondria, membranes, ribosomes, microtubules, and open space outside of membranes (Figure S3).[25] This substantial open space between cells contrasts with chemically fixed tissues, where a large portion of the extracellular space is lost due to chemical fixation. Similar observations have also been made for tissue that has undergone cryo-fixation—without consideration for vitrification—compared to chemical fixation.[25] Ultimately, our vitrification strategy did not appear to be detrimental to cellular features, suggesting its compatibility with *in situ* cryo-ET experiments.

Hippocampal layer targeting using cryo-correlative light and electron microscopy

Using the known architecture of mouse hippocampal layers, we targeted CA1-sp and CA1-sr by mapping the locations of cell bodies with the live-cell nuclear stain Hoechst (Figures 1B and 1C). Cryo-confocal microscopy overviews (Figure 1B) allowed screening and the orientation of biopsies prior to mapping with the integrated (wide-field) fluorescence microscope in the FIB/SEM. Orientation information was subsequently used to position the carrier for milling and fluorescence correlation for targeted lift-out (Figure 1C). To map the cellular organization of the entire tissue section, we acquired fluorescence tilesets that spanned the

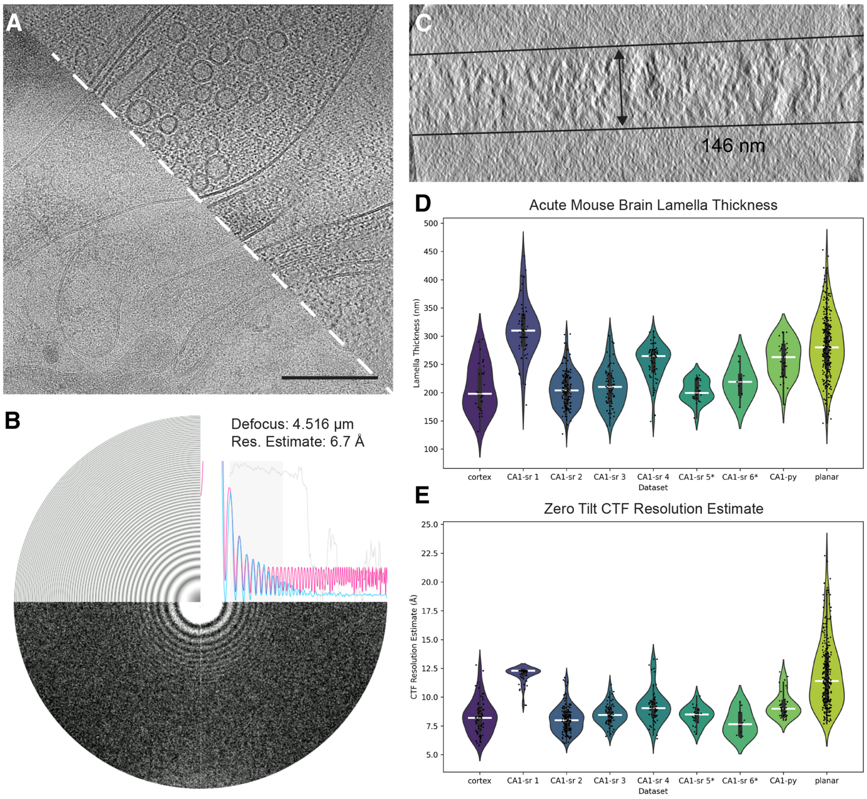

Figure 2. Quality of cryo-ET data from lifted-out mouse brain

(A) Raw 0° tilt image before preprocessing motion correction (bottom left) and after reconstruction (top right). Scale bar: 200 nm.

(B) Contrast transfer function experimental (blue) and estimated (pink) functions, defocus, and resolution estimates calculated in the Warp software package.[23]

(C) Reconstructed tomogram thickness measured in IMOD.[24]

(D) Thickness measurements from all tomograms in the 9 vitreous datasets acquired in this work. The cryoprotectant used for all datasets was kept consistent, except for datasets with an *, where CA1-sr 5 was frozen in 10% dextran, 10% sucrose in artificial CSF (aCSF) (pH 7.4) and CA1-sr 6 was frozen in 10% dextran, 5% sucrose, 5% ethylene glycol in aCSF (pH 7.4).

(E) CTF resolution estimate from the 0° tilt output from Warp during initial image processing.

entire 2 × 2 mm carrier. With a 20× fluorescence objective, these tilesets could be acquired in as little as 20 min for one fluorophore, depending on the desired number of Z steps (Table S2).

We initially targeted, lifted out, sectioned, and thinned regions of tissue containing fluorescently labeled nuclei from CA1-sp (Figure 3), as this enabled us to validate our approach for fluorescent targets. Individual nuclei, including regions of heterochromatin and euchromatin, could be identified within the cryo-fluorescence data (Figures 3A–3C). Retention of fluorescent targets could be monitored at all stages, from initial carrier overviews and z stacks through to the final thinned lamellae, to aid in targeted tilt series acquisition in TEM. In this hippocampal layer, we were able to collect tilt series where most of the field of view consisted of ribosomes along with tomograms containing mitochondria and vesicles, in line with features expected within and near cell bodies (Figure 3E).

Beyond CA1-sp, we were also able to target sublayers that were largely devoid of nuclei. In this case, we selected CA1-sr. This layer is known to be rich in synaptic connections[26] and can be targeted via proximity to CA1-sp, which was readily identified when CA3-sp and dentate gyrus were present in slices (Figure 1B). From this sublayer, we collected six datasets originating from 2 mice, one male and one female, totaling 359 tilt series (Table S3). In contrast to the CA1-sp datasets, we did not observe large numbers of ribosomes. As specific mechanisms exist to keep ribosomes localized to cell bodies rather than migrating along neuronal processes, this is consistent with characteristics expected of CA1-sr. Instead, we observed cellular features more typically observed in neuronal processes. These included mitochondria with (13/359, or 4%) and without (119/359, or 33%) granular deposits[27,28]; microtubules (302/359, or 84%) and thinner filaments including actin (114/359, or 32%) in dendrites and other processes; small clusters of 1–20 ribosomes (60/359, or 17%); and processes with vesicles (311/359, or 87%) and synapses (56/359, between 3% and 30% of tomograms collected from each dataset) (Figures 1G, 4, S4, and S5; Table S3). For many of these synapses, cell adhesion molecules and inter-membrane interactions could be seen (Figures 1G and 2).

Synapse organization within CA1-sr

Synapses could be identified within tomograms by their canonical organization, consisting of pre- and postsynaptic compartments separated by a cleft (Figure 5A). The presynaptic compartment consisted of vesicles and, in some cases, mitochondria, while actin filaments, small clusters of ribosomes, and membrane-associated densities could be observed in the postsynaptic compartment (Figure S5).

Synaptic vesicles appeared spherical, with a mean diameter of 45.4 ± 2.2 nm (Figures 4 and S6), consistent with previously reported values for cryogenically preserved synaptic vesicles[29,30] and larger than vesicles in resin-embedded synapses.[31] We did not detect any statistically significant variation in synaptic vesicle size as a function of postmortem interval (Figure S6G).

We were able to observe synaptic vesicle fusion with the presynaptic membrane in some instances of our data (8/107, 7%) (Figures 5B and 5C), evidencing synaptic activity occurring at the time of freezing. We observed all stages of fusion (Figure 5C), including initial vesicle tethering to the presynaptic membrane via densities[30] that could be recruitment of molecules such as Munc13-1[32] (Figures 5C and 5C1). We could also distinguish large vesicles with dense cores from synaptic vesicles, which could be identified by their size and electron-dense interior[28] (Figure S5).

CellPress
OPEN ACCESS

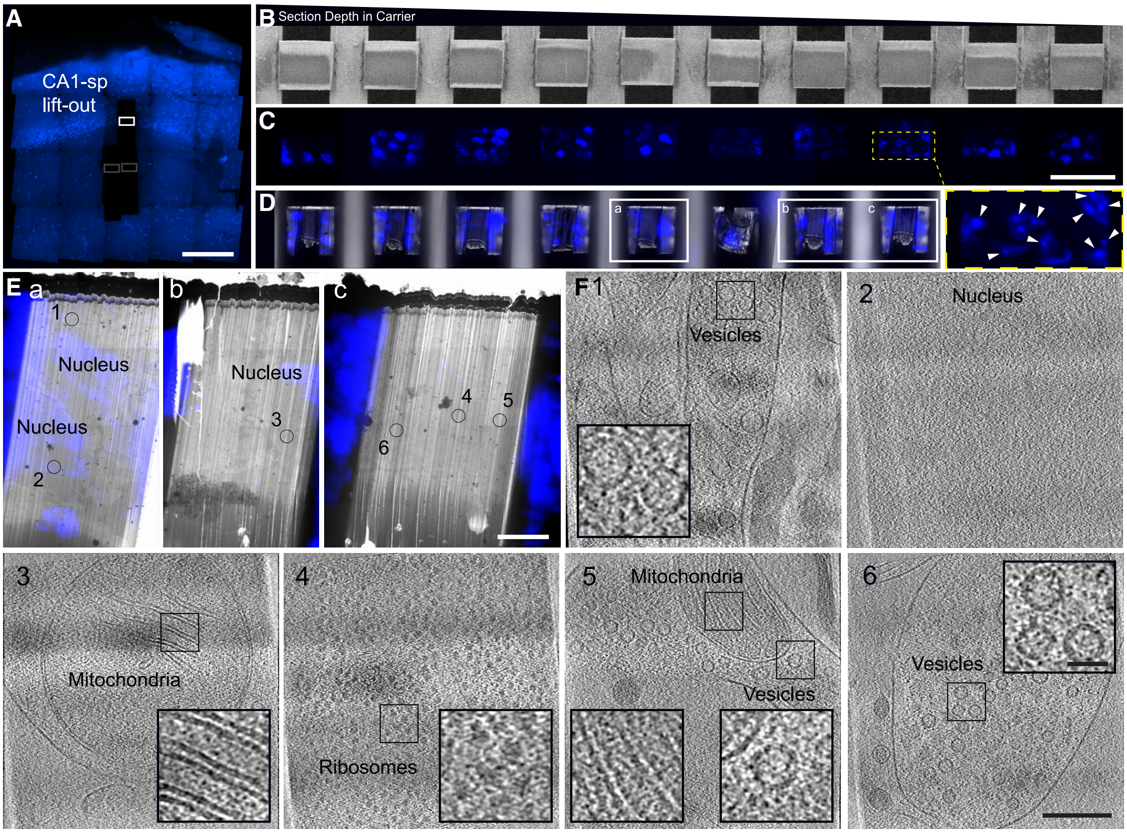

Figure 3. Fluorescence targeting and observable features in CA1-sp

(A) Fluorescence tile set of the same carrier used in Figure 1 showing the lift-out positions for CA1-sr (gray boxes) and CA1-sp (white box). Fluorescence image was acquired using an integrated fluorescence module (Delmic Meteor) on the Helios Hydra PFIB/SEM. Scale bar: 200 μm.

(B) SEM images of serially deposited sections from CA1-sp lift-out.

(C) Fluorescence images of serially deposited sections from CA1-sp lift-out. Inset highlights diffuse fluorescence indicative of euchromatin, and more punctate fluorescence (arrows) highlights heterochromatin. Fluorescence image was acquired using an integrated fluorescence module (Delmic Meteor) on the Helios Hydra PFIB/SEM. Scale bar (B)–(D): 50 μm.

(D) Thinned lamella with fluorescence overlay in SEM. Fluorescence images were acquired using an integrated fluorescence module on the Arctis PFIB/SEM.

(E) Lamellae boxed in (D) (a–c) where fluorescence and TEM overviews are overlaid and select tilt series acquisition positions marked 1–6. Scale bar: 5 μm.

(F) Slices through reconstructed tomograms acquired at positions 1–6. Position 2 was acquired in a fluorescent, nuclear region of the lamella. Insets highlight vesicles, mitochondria cristae, and ribosomes. Scale bar for tomograms: 200 nm; scale bar for insets: 40 nm.

The synaptic cleft consists of two closely mated membranes with cell adhesion molecules spanning the 20–30 nm[28] between the compartments. In some instances, molecules on the postsynaptic face were not in contact with partners on the presynaptic face (Figure 4). In the postsynaptic compartment, we were often able to observe actin filaments. Likewise, we found small clusters of ribosomes only on the postsynaptic side (Figure S5), which is consistent with ribosomes being more common in dendrites compared to axons. In some tomograms, we could identify a region near the membrane that appeared more electron dense, indicative of a putative postsynaptic density (PSD) (Figure S5). However, importantly, these could not always be observed (Figure S5).

Assessment of the molecular organization of tissue from CA1-sp to CA1-sr

Serial lift-out allows for semi-continuous sampling across an organism or tissue. By utilizing the faster milling rate of the PFIB, previously unattainable regions can be accessed, as more material can be excavated in a shorter time. When observed from different perspectives, aspects of the neuronal organization previously unseen by cryo-ET can be revealed. To complement our existing data, we developed a strategy to investigate morphological features of CA1-sr that run within the plane of the sample (Figures 4A and S1).

Using cryo-lift-out, we excised regions that span CA1-stratum oriens (CA1-so) to lacunosum moleculare (CA1-slm), investigating how constituents change across hippocampal sublayers. Hippocampi were mapped using cryo-correlative light and electron microscopy (cryo-CLEM) as previously outlined (Figure 1), identifying a region containing multiple layers of CA1. Trenches were milled using 200 nA Xe around the region of interest (Figures 6B and 6C). In two replicates, the regions lifted out were 350 and 390 μm in length, yielding 40 and 42 sections of ~5 μm in thickness, which were amenable to lamella fabrication (Figures 6D and 6E). Serial sections

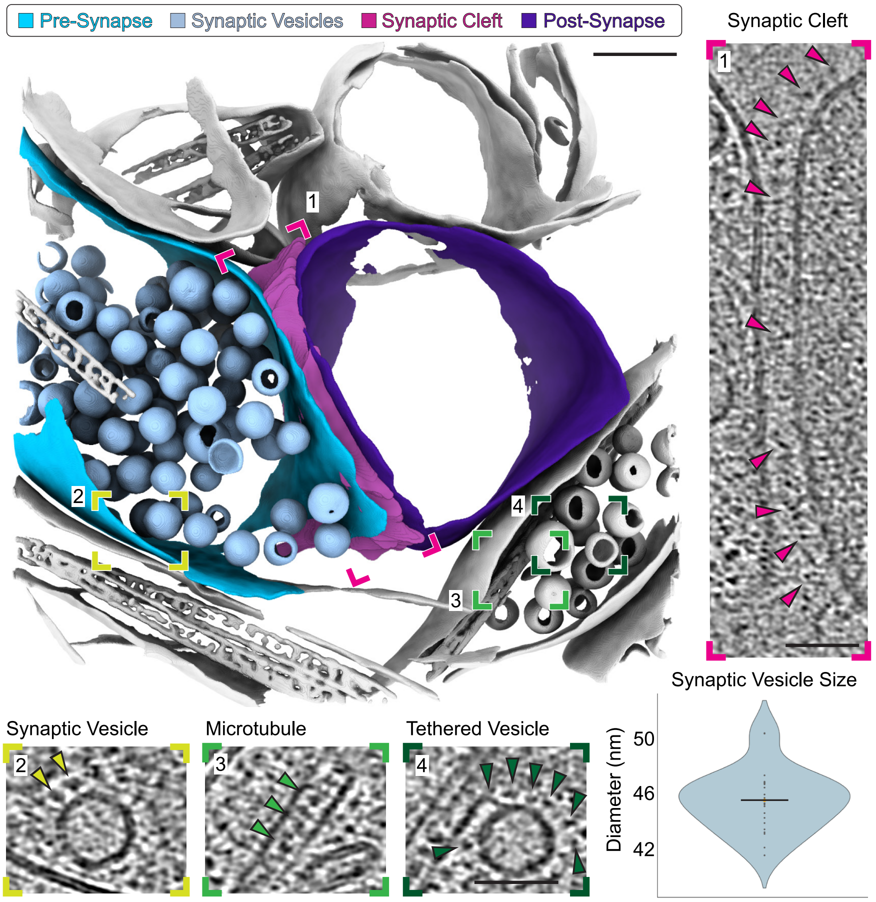

Figure 4. Molecular features of a CA1-sr synapse

Segmentation of a synapse with synaptic vesicles (light blue), synaptic cleft (pink), presynaptic membrane (blue), and postsynaptic membrane (purple) highlighted. Scale bar: 100 nm. Insets from the deconvolved tomogram highlight details like synaptic cleft densities (right, pink arrows), synaptic vesicles with membrane-attached densities (yellow, left and dark green, right arrows), and microtubules with interior densities (middle green arrows). Scale bar in insets: 50 nm. The distribution of synaptic vesicle diameters across a subset of 27 tomograms where the only vesicles present are in a visible synapse (bottom right) is shown. Each dot represents the per-tomogram mean of the median of the per-slice 2D Feret diameter of each vesicle.

from one of these planar lift-outs were taken forward, where 39 lamellae were thinned and transferred to the TEM for tilt series acquisition. From 13 imaged lamellae, 246 tomograms were generated spanning a 150 μm region directly below CA1-sp.

From these data, the molecular composition across CA1-sr was characterized. Within cells, typical features were identified at each distance across the serial lift-out. These included both in-plane and perpendicular microtubules (221/246, or 90%) and thinner filaments, including actin (75/246, or 30%) and smooth endoplasmic reticulum (ER) (38/246, or 15%) (Figure 6G).

By isolating tissue from within the plane of the sample, we could observe CA1 apical dendrites from an axial perspective (30/246, or 12%) (Figure 6G). This view would be inaccessible when milling cells on grids since sections can only be produced obliquely to the plane of the neuronal processes. Dendrite cross-sections can be readily identified from medium-magnification TEM images for targeted tilt series acquisition by the dense arrangement of microtubules (Figure 6F). In contrast, perpendicular lift-outs in the same region of CA1-sr did not yield many apical dendrite cross-sections where head-on views of microtubules and actin could be found (1/359, or 0.3%) but instead favored an orientation with side views of dendrites where groups of microtubules could be found traversing the field of view (10/359, or 3%) (Figure S4B).

Analysis of the apical dendrite cytoskeleton network from planar lift-out

A representative segmentation depicting the axial view of a dendrite (seen in 30 tomograms) illustrates the main cellular components observed (Figure 7). The most common features were axial microtubules, extruded ER, and bundles of actin filaments, which were found in every tomogram (Figures 7 and S7A). Less frequent observations included dense, matrix-like patches (9/30), mitochondria (7/30), and double-membraned vesicles of various sizes (8/30) (Figure S7A).

The number of microtubules observed in dendritic cross-sections was examined in relation to their distance from CA1-so (Figure S7B). Interfilament distances had a mean value of 57.7 ± 22.4 nm (mean ± SD) and a median of 54.0 nm (n = 481). The majority of the filaments were spaced between 40 and 70 nm, with less frequent interfilament spacing ranging beyond 200 nm (Figure S7C). This suggests there is predominantly regular spacing between filaments, with occasional large spacings, possibly to accommodate intracellular cargo trafficking of organelles. The spacing did not change significantly as a function of distance across the lift-out, suggesting no trend in interfilament distance between layers of CA1 (Figure S7D).

Microtubules of both 13 and 14 protofilament (pf) originating from dendrites could be observed (Figures 7B and 7C), with lattice defects in 3% (15/481) of filaments. In 15/18 of the defective filaments, there were one or more protofilaments missing throughout the volume, and in 3/18, there were incomplete ends (Figure 7D). Density was also visible within the lumenal space, with additional density connecting lumenal particles and the inner microtubule wall (Figures 7E and 7F). Interestingly, bridging densities can be seen between some microtubules in proximity, suggesting that at least some microtubules are linked by putative tethers

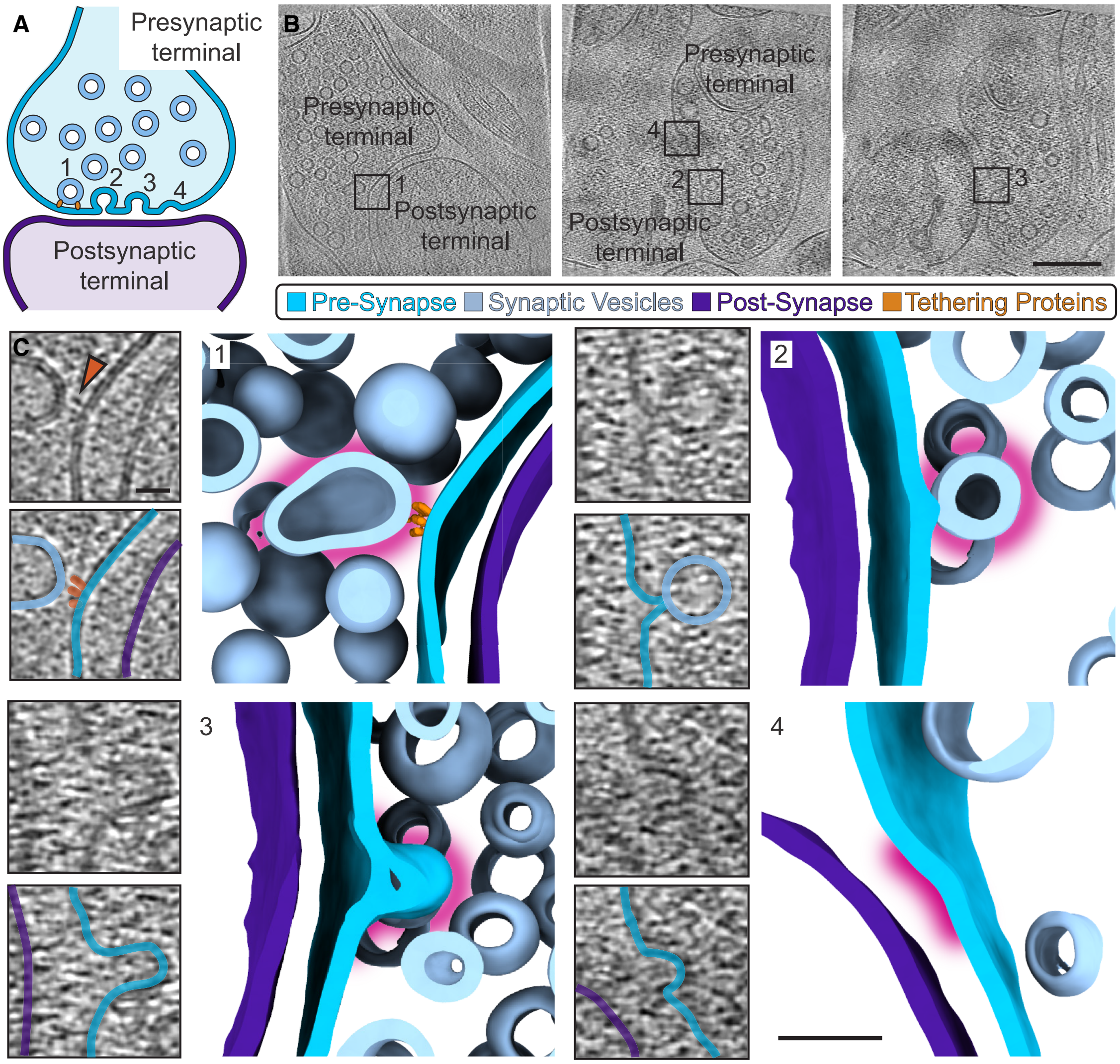

Figure 5. Synaptic vesicles fusing with the presynaptic membrane

(A) Cartoon of synapse highlighting presynaptic terminal and membrane (electric blue), synaptic vesicles (light blue), postsynaptic terminal and membrane (purple), and proteins tethering synaptic vesicles ready for fusion with the presynaptic membrane (orange).

(B) Slices through two tomograms with synaptic vesicle fusion events. Scale bar: 200 nm.

(C) Zoom-in vesicles at different stages of fusion with the presynaptic membrane and corresponding segmentations. Pink halo highlights the vesicle or curved portion of the membrane of interest. Scale bar in tomogram: 20 nm; scale bar in segmentation: 50 nm.

(Figure 7G). The microtubules also interact with the ER, mediated by multiple tethering interactions (Figure 7H).

DISCUSSION

Here, we demonstrate an approach for the targeting of specific regions of brain tissue for molecular imaging. Notably, this did not require genetically modified animals with fluorescently encoded tags or injection of material, illustrating that with generic fluorescent labeling, a diverse range of experimental setups, tissues, and animal models of varying genetic backgrounds can be assessed. Presently, our data represent a benchmark for assessing brain molecular organization in CA1-sr within the C57BL/6 inbred mouse.

Brains were dissected and slices vitrified 1.5–4 h postmortem, which is a time frame consistent with other studies.[33,34] The main factors affecting this range of intervals is the time taken to sacrifice, prepare, section, incubate with cryoprotectant, and then high-pressure freeze 100–200 μm sections of brain. In previous studies that utilized CEMOVIS, time for incubation post-sectioning was reported,[35,36] but total time postmortem was not, nor were the range of intervals used postmortem up to vitrification. In our experience, brain slices kept in similar buffer conditions and cultured at a postmortem interval <3 h are viable in organotypic culture experiments. This supports the conclusion that acute slices prepared as we described in this manuscript would retain some level of functionality prior to cryoprotectant incubation but are not expected to retain full functionality.[33,34] The

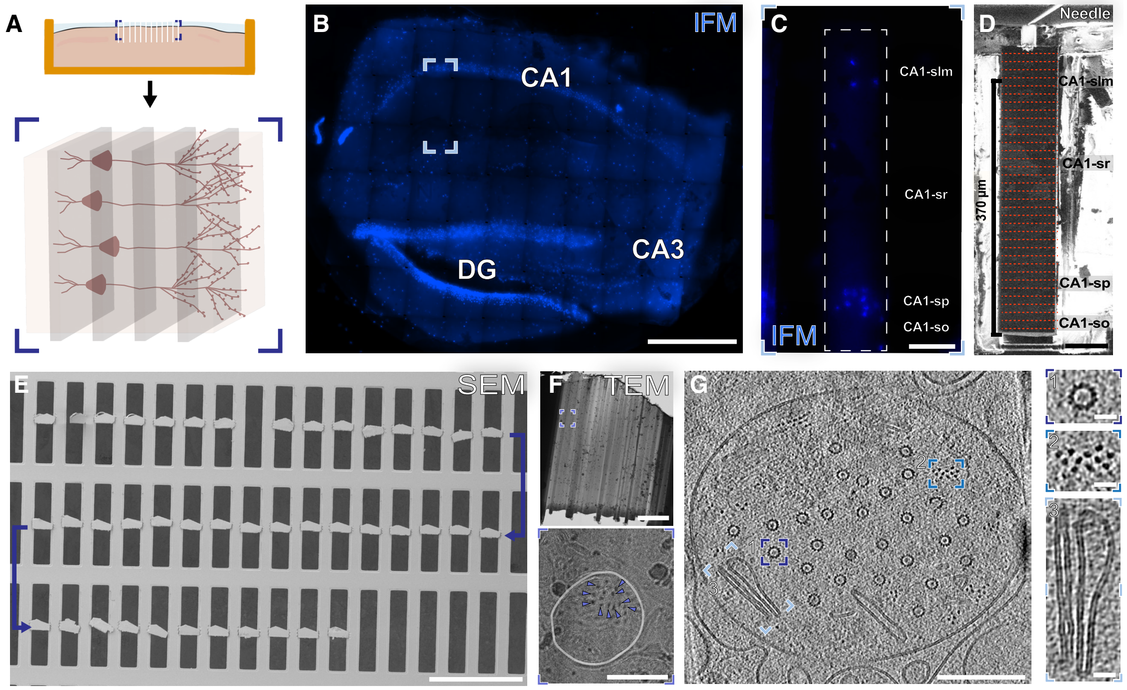

Figure 6. Planar lift-out of CA1 strata oriens to lacunosum moleculare

(A) (Top) Schematic of a high-pressure-frozen hippocampal section depicted as a cross-section, with the region for planar lift-out (blue box) and serial sections (white lines) highlighted. (Bottom) The orientation of the pyramidal cells in the planar lift-out sections are shown, with gray slabs exemplifying serial sections capturing different slices through connected tissue.

(B) IFM image of a hippocampal section used for planar lift-out (blue box) with CA1, CA3, and DG marked. Scale bar: 200 µm.

(C) Fluorescence image of the lift-out target depicted in (B) after trench milling, with sub-regions labeled. Scale bar: 50 µm.

(D) FIB image of the 370 µm lift-out target with needle attached and marked with rough locations of serial sections to be subsequently deposited for lamella fabrication. Scale bar: 50 µm.

(E) SEM image of all 42 deposited serial sections. Arrow denotes direction of deposition, starting from CA1-so in the top left through CA1-slm in the bottom right. Scale bar: 200 µm.

(F) (Top) A medium-magnification TEM image of a representative lamella from CA1-sr. Scale bar: 5 µm. (Bottom) Magnified region of (F) at inset indicated above (blue) shows a dendritic cross section (plasma membrane highlighted in white) with microtubules (blue arrows). Scale bar: 1 µm.

(G) 2D slice through a reconstructed dendrite tomogram depicting (1) head-on views of microtubules, (2) bundles of actin filaments, and (3) extruded endoplasmic reticulum. Scale bar: 200 nm; scale bar for insets: 25 nm.

practical steps required to prepare and perform each vitrification mean that short postmortem intervals remain challenging. While future attempts to integrate fast-freezing approaches from volume EM into cryo-ET-enabled workflows[33] could have tangible benefits on the visualization of processes, reducing the postmortem interval would require the development of a novel vibratome to high-pressure-freezer fast-freezing method.

The characterization of cryoprotectants and their ability to enable vitrification began with the use of combinations that had previously been reported for the vitrification of thick specimens[37,38] while also maintaining tissue health.[39] We incorporated three buffers—phosphate buffer, artificial cerebral spinal fluid (CSF), and N-methyl-D-glucamine (NMDG)-substituted artificial CSF[40]—into our cryoprotectant formulation, demonstrating that similar results can be achieved using a range of typical physiological buffers (Table S1). The requirement of low-MW cryoprotectants such as sucrose and an incubation period with the sample means that there will be osmolarity changes in the tissue, affecting its physiology. This will need to be carefully considered when designing experiments. Ultimately, the sample preservation presented here permits visualization of individual proteins from the resulting tomograms. This opens up the potential for subtle structural changes that could underpin the developmental or disease mechanisms to be observed.

We demonstrate uncompressed preservation of tissue morphology and complete vitrification by HPF, where hippocampal layers could be easily identified by CLEM post-freezing (Figures 1B, 1C, and 6B). In previous approaches where only very thin specimens could be frozen on the grid,[21] the brain was exposed to large forces against grid bars, causing distortions of tissues, which limits sub-region targeting. HPF has been used to generate brain samples for subsequent analysis by cryo-ET but has been limited to very few ($<N = 2$) examples, with no thorough assessment of vitrification.[13,15,35,41,42] In the present study, the method of freezing allows viable tissue slices to be frozen in a format where samples are protected from compression during freezing and vitrification is reproducible and robust.

For FIB milling, the use of xenon plasma was particularly advantageous. Large trenches on the order of 500,000 μm^3 of material—100× the volume of a HeLa cell—can be cleared in under

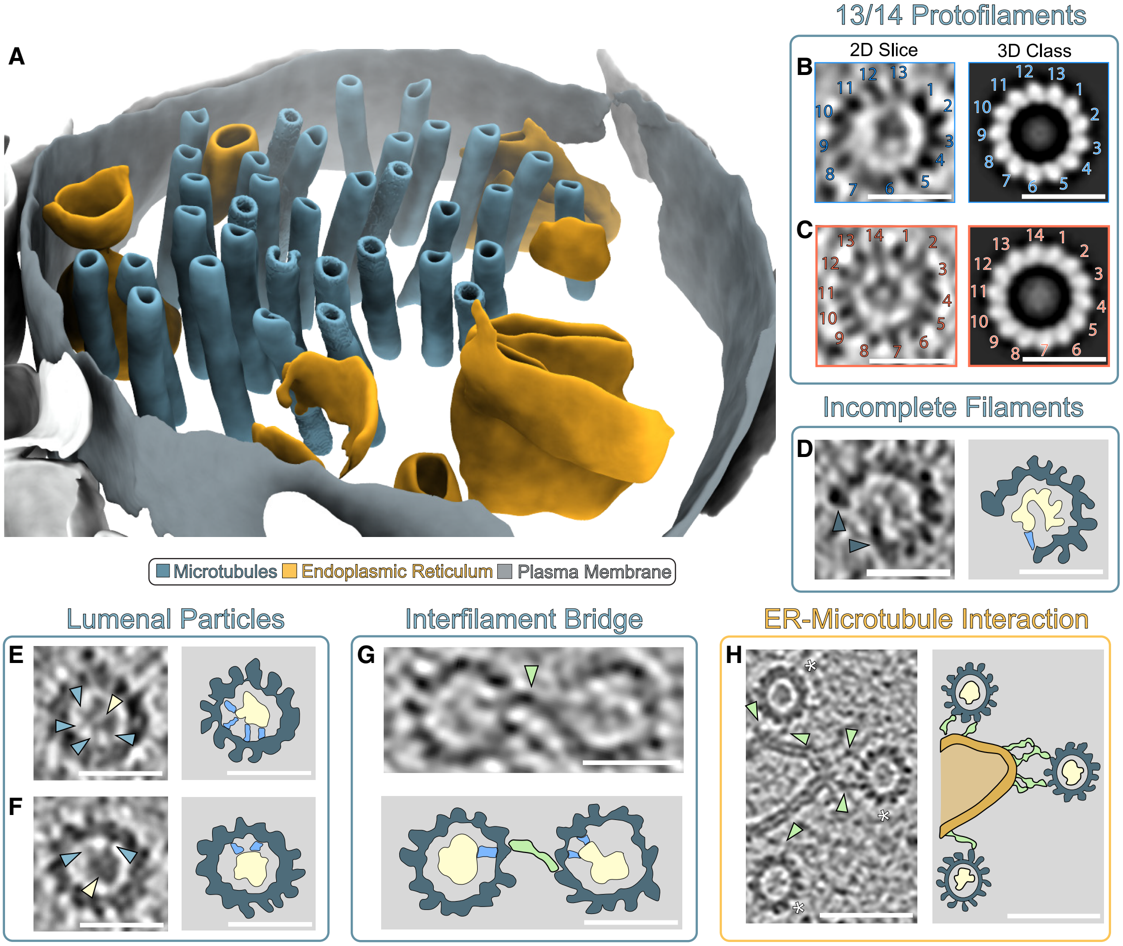

Figure 7. Characterization of the apical dendrite network cytoskeleton from planar lift out

(A) Segmentation of a representative tomogram depicting a cross-section of a dendrite with microtubules (blue), with the endoplasmic reticulum (gold) surrounded by the plasma membrane (gray) highlighted.

(B and C) 2D slices of reconstructed tomograms and 3D classifications of microtubules composed of both 13 (blue box) and 14 (orange box) pf, respectively. Scale bar: 25 nm.

(D) An example of an incomplete microtubule devoid of multiple protofilaments, with density seen connecting the edge of the microtubule to an internal density (blue). Scale bar: 25 nm.

(E and F) Examples of lumenal particles within microtubules (yellow) with multiple associated tethers to the inner microtubule wall (blue). Scale bar: 25 nm.

(G) Example of two microtubules interacting through a bridging density (green). Scale bar: 25 nm.

(H) Example of the interactions between microtubules (*) and the endoplasmic reticulum (orange) linked via bridging densities (green). Scale bar: 50 nm.

30 min to isolate material from an HPF carrier using undercuts >50 μm below the sample surface. Traditional LMIS Ga^+ FIBs are significantly slower,[17,20] meaning the removal of large amounts of material with gallium is impractical. The speed demonstrated here also negates the need for extra cryo-microtomy steps to remove excess material from the tissue and carrier.[13,35,42–44] Consistent with the thinning of cellular lamellae,[17,18,45] we were also able to generate electron-transparent lamellae from tissues using a PFIB (Figure 2). This demonstrates the versatility of plasma for clearing large volumes at speed with high currents without sacrificing sample quality during thinning at low currents.[17,20,45]

Crucially, the strength of our approach is the ability to image molecules within hippocampus. As a focus, we aimed to image in CA1-sr, a region rich in synapses, including Schaffer collaterals.[23,46] Our data are consistent with this, with multiple examples of active synaptic boutons and the absence of myelinated axons. The resulting data were of sufficient quality to visualize synaptic vesicle- and synaptic cleft-spanning proteins. For diameter, a large range of vesicle sizes was found, ranging from 41.2 to 51.2 nm as an average per tomogram. We did not find any statistically significant differences in vesicle diameter as a function of the sex of the mouse, cryoprotectant and buffer composition, postmortem interval before vitrification, or dataset (Figure S6; Table S3). As vesicles suffer from missing wedge artifacts, volume determination will always require some missing wedge correction inferences or smaller estimates of volume and lower measures of

sphericity than may be true in reality if no interpolation in the form of missing wedge correction is made.

The ability to extract tissue samples in a variety of orientations allows for lamellae to be fabricated with previously unobtainable views through conventional cellular cryo-ET experiments. The orientation of sectioning could, in theory, be altered depending on the view required by the operator, but incorporating alternative orientations for lift-out as performed here enables the ability to orient biological features for imaging from multiple angles from the same sample. This reduces the detrimental effect of artifacts arising from the missing wedge of information in Fourier space. Furthermore, as hippocampus is a differentiated tissue, cell populations are structured in an organized fashion, where features of interest exist in specific planes. In the present case, dendrites originating from CA1-sp run parallel to one another, traversing our sample plane in CA1-sr. By preserving the native, hydrated state of the dendrite, fine molecular details that are obfuscated by chemical fixation could be observed (Figure 7). As we integrate multi-scale correlation, the spatial relationship of sublayers within the tissue with respect to the bulk sample can be maintained and tracked between sequentially deposited sections. Tissue composition, such as cell and synapse types, has been shown to vary from regions of CA1-sr more proximal to CA1-sp to more distal regions.[47] The ability to sample every 5–10 μm facilitates investigation of the subtle changes in molecular composition within and between layers.

In summary, our work demonstrates a robust and flexible strategy for the preparation of frozen-hydrated, natively preserved (brain) tissue. Importantly, scaling structural biology to tissues requires techniques that can generate enough samples, in a targeted manner, that allow a pool of tomograms from cohorts of individuals. This requires reproducibility, a high success rate, and feasible time frames. We were able to generate tens of tissue chunks per day from individual slices, which incorporate large-scale mapping from the millimeter to the nanometer scale. Our data demonstrate pristine preservation, allowing an analysis of molecular context within the brain. This lays the framework for the routine assessment of tissue samples in the future, where specific features relating to pathology may be correlated and assessed on a molecular scale. Crucially, this allows for an assessment of different samples in the order of days, potentially enabling targeted clinical observations at scale. Future work will incorporate the integration of scanning electron methods as well as machine learning to improve automation and feature recognition.

Limitations of the study

To vitrify the tissue biopsies, it was necessary to incubate samples in cryoprotectant for a period of time. While leading to complete vitrification, the physiological effects of this treatment are not investigated, and therefore, the impact of this on specific biological applications moving forward would need to be ascertained. In addition, a generic live-cell stain is used to target sublayers within mammalian hippocampus for cryo-ET investigation. While useful for broad targeting to investigate sub-regions, aspects such as cell types within sublayers cannot be determined without more specific markers. Finally, the serial cryo-lift-out approach used offers semi-continuous sampling but leads to inherent loss of information between serial sections, as material must be removed to achieve electron transparency. This material loss means that features cannot be followed between sections easily, especially when compared with analogous volumetric methods such as serial block-face EM. Future integration of volume electron microscopy approaches, coupled with approaches for more fine sectioning of material during sectioning, would increase the ability to trace the molecular organization across striated populations of cells.

RESOURCE AVAILABILITY

Lead contact

Requests for further information and resources should be directed to and will be fulfilled by the lead contact, Michael Grange (michael.grange@rfi.ac.uk).

Materials availability

No new materials or reagents were generated in this study.

Data and code availability

All microscopy data reported in this paper will be shared by the lead contact upon request. The segmentation software used for vesicle segmentation is the Python package Volume Segmantics, which is available at https://github.com/rosalindfranklininstitute/volume-segmantics.[48] The model trained for segmentation with Volume Segmantics is available at https://doi.org/10.5281/zenodo.15322367. The vesicle measurements were conducted using the code available at https://github.com/rosalindfranklininstitute/vesicle_measurement. Any additional information required to reanalyze the data reported in this paper is available from the lead contact upon request.

ACKNOWLEDGMENTS

We would like to thank Dr. Sara Wells, Dr. Marianne Yon, Dr. Michelle Stewart, and Jessica Podd from the Mary Lyon Centre for Mouse Genetics at MRC Harwell for their support in animal work. We would like to thank Dr. Victoria Garcia-Giner for assistance acquiring cryo-confocal images. We would also like to thank Dr. Casper Berger, Dr. Charlie Lovatt, and Helena Watson for their assistance with data processing and analysis. This work was supported by a Wellcome Career Development Award (225902/Z/22/Z to M.G.) and through the Wellcome-funded "Electrifying Life Science" grant (220526/Z/20/Z to Prof. James H. Naismith). J.L.R.S. is supported by a Wellcome Trust PhD Studentship (226810/Z/22/Z). The Rosalind Franklin Institute is funded by UK Research and Innovation through the Engineering and Physical Sciences Research Council (EPSRC).

AUTHOR CONTRIBUTIONS

M.G. conceptualized the work. R.C. and M.E.S. performed vibratome slicing and hippocampal dissection. C.G. and J.L.R.S. carried out HPF. J.L.R.S. and C.G. optimized the initial lift-out with input from R.C. and T. S.G. C.G. collected cryo-confocal images. M.C. and C.G. developed the targeting approach for lift-out. C.G. and J.L.R.S. carried out serial lift-out and FIB/SEM experiments. C.G., M.C., and J.L.R.S. collected cryo-ET data. C.G. and J.L.R.S. reconstructed tomograms. A.K. and A.P. performed segmentation and analysis of synaptic vesicles. C.G., J.L.R.S., and M.G. analyzed the data. J.L.R.S. and M.G. performed sub-volume averaging. The initial draft was written by C.G. and J.L.R.S. and edited by C.G., J.L.R.S., and M.G. with input from all authors. Funding was acquired by M.G.

DECLARATION OF INTERESTS

The authors declare no competing interests.

STAR★METHODS

Detailed methods are provided in the online version of this paper and include the following:

- KEY RESOURCES TABLE
- EXPERIMENTAL MODEL AND STUDY PARTICIPANT DETAILS
  - Animal handling and brain dissection
- METHOD DETAILS
  - Sectioning of acute brain slices
  - High-pressure freezing
  - Cryo-confocal microscopy
  - Hippocampal layer targeting
  - Cryo-lift-out
  - Serial section deposition
  - Fine milling of serial lift-out sections
  - Cryo-electron tomography tilt series acquisition
  - Tomogram reconstruction
  - Tomogram segmentation
  - Filament analysis
  - Sub-volume averaging
- QUANTIFICATION AND STATISTICAL ANALYSIS

SUPPLEMENTAL INFORMATION

Supplemental information can be found online at https://doi.org/10.1016/j.crmeth.2025.101080.

Received: December 3, 2024
Revised: March 6, 2025
Accepted: May 19, 2025
Published: June 16, 2025

REFERENCES

1. Medalia, O., Weber, I., Frangakis, A.S., Nicastro, D., Gerisch, G., and Baumeister, W. (2002). Macromolecular Architecture in Eukaryotic Cells Visualized by Cryoelectron Tomography. Science *298*, 1209–1213. https://doi.org/10.1126/science.1076184.
2. Nicastro, D., McIntosh, J.R., and Baumeister, W. (2005). 3D structure of eukaryotic flagella in a quiescent state revealed by cryo-electron tomography. Proc. Natl. Acad. Sci. *102*, 15889–15894. https://doi.org/10.1073/pnas.0508274102.
3. Lučić, V., Leis, A., and Baumeister, W. (2008). Cryo-electron tomography of cells: connecting structure and function. Histochem. Cell Biol. *130*, 185–196. https://doi.org/10.1007/s00418-008-0459-y.
4. Michel, M., Hillmann, T., and Müller, M. (1991). Cryosectioning of plant material frozen at high pressure. J. Microsc. *163*, 3–18. https://doi.org/10.1111/j.1365-2818.1991.tb03155.x.
5. Richter, K. (1994). Cutting artefacts on ultrathin cryosections of biological bulk specimens. Micron *25*, 297–308. https://doi.org/10.1016/0968-4328(94)90001-9.
6. Hsieh, C.-E., Marko, M., Frank, J., and Mannella, C.A. (2002). Electron tomographic analysis of frozen-hydrated tissue sections. J. Struct. Biol. *138*, 63–73. https://doi.org/10.1016/S1047-8477(02)00034-5.
7. Al-Amoudi, A., Dubochet, J., Gnaegi, H., Lüthi, W., and Studer, D. (2003). An oscillating cryo-knife reduces cutting-induced deformation of vitreous ultrathin sections. J. Microsc. *212*, 26–33. https://doi.org/10.1046/j.1365-2818.2003.01244.x.
8. Al-Amoudi, A., Studer, D., and Dubochet, J. (2005). Cutting artefacts and cutting process in vitreous sections for cryo-electron microscopy. J. Struct. Biol. *150*, 109–121. https://doi.org/10.1016/j.jsb.2005.01.003.
9. Marko, M., Hsieh, C., Moberlychan, W., Mannella, C.A., and Frank, J. (2006). Focused ion beam milling of vitreous water: prospects for an alternative to cryo-ultramicrotomy of frozen-hydrated biological samples. J. Microsc. *222*, 42–47. https://doi.org/10.1111/j.1365-2818.2006.01567.x.
10. Marko, M., Hsieh, C., Schalek, R., Frank, J., and Mannella, C. (2007). Focused-ion-beam thinning of frozen-hydrated biological specimens for cryo-electron microscopy. Nat. Methods *4*, 215–217. https://doi.org/10.1038/nmeth1014.
11. Rigort, A., Bäuerlein, F.J.B., Villa, E., Eibauer, M., Laugks, T., Baumeister, W., and Plitzko, J.M. (2012). Focused ion beam micromachining of eukaryotic cells for cryoelectron tomography. Proc. Natl. Acad. Sci. USA *109*, 4449–4454. https://doi.org/10.1073/pnas.1201333109.
12. Villa, E., Schaffer, M., Plitzko, J.M., and Baumeister, W. (2013). Opening windows into the cell: focused-ion-beam milling for cryo-electron tomography. Curr. Opin. Struct. Biol. *23*, 771–777. https://doi.org/10.1016/j.sbi.2013.08.006.
13. Nguyen, H.T.D., Perone, G., Klena, N., Vazzana, R., Kaluthantrige Don, F., Silva, M., Sorrentino, S., Swuec, P., Leroux, F., Kalebic, N., et al. (2024). Serialized on-grid lift-in sectioning for tomography (SOLIST) enables a biopsy at the nanoscale. Nat. Methods *21*, 1693–1701. https://doi.org/10.1038/s41592-024-02384-6.
14. Kelley, K., Raczkowski, A.M., Klykov, O., Jaroenlak, P., Bobe, D., Kopylov, M., Eng, E.T., Bhabha, G., Potter, C.S., Carragher, B., and Noble, A.J. (2022). Waffle Method: A general and flexible approach for improving throughput in FIB-milling. Nat. Commun. *13*, 1857. https://doi.org/10.1038/s41467-022-29501-3.
15. Creekmore, B.C., Kixmoeller, K., Black, B.E., Lee, E.B., and Chang, Y.-W. (2024). Ultrastructure of human brain tissue vitrified from autopsy revealed by cryo-ET with cryo-plasma FIB milling. Nat. Commun. *15*, 2660. https://doi.org/10.1038/s41467-024-47066-1.
16. Dumoux, M., Glen, T., Smith, J.L.R., Ho, E.M.L., Perdigão, L.M.A., Pennington, A., Klumpe, S., Yee, N.B.Y., Farmer, D.A., Lai, P.Y.A., et al. (2023). Cryo-plasma FIB/SEM volume imaging of biological specimens. eLife *12*, e83623. https://doi.org/10.7554/eLife.83623.
17. Berger, C., Dumoux, M., Glen, T., Yee, N.B. -y, Mitchels, J.M., Patáková, Z., Darrow, M.C., Naismith, J.H., and Grange, M. (2023). Plasma FIB milling for the determination of structures in situ. Nat. Commun. *14*, 629. https://doi.org/10.1038/s41467-023-36372-9.
18. Rangan, R., Khavnekar, S., Lerer, A., Johnston, J., Kelley, R., Obr, M., Kotecha, A., and Zhong, E.D. (2023). Deep reconstructing generative networks for visualizing dynamic biomolecules inside cells. Preprint at bioRxiv. https://doi.org/10.1101/2023.08.18.553799.
19. Kelley, R., Khavnekar, S., Righetto, R.D., Heebner, J., Obr, M., Zhang, X., Chakraborty, S., Tagiltsev, G., Michael, A.K., Dorst, S. van, et al. (2024). Towards community-driven visual proteomics with large-scale cryo-electron tomography of Chlamydomonas reinhardtii. Preprint at bioRxiv. https://doi.org/10.1101/2024.12.28.630444.
20. Burnett, T.L., Kelley, R., Winiarski, B., Contreras, L., Daly, M., Gholinia, A., Burke, M.G., and Withers, P.J. (2016). Large volume serial section tomography by Xe Plasma FIB dual beam microscopy. Ultramicroscopy *161*, 119–129. https://doi.org/10.1016/j.ultramic.2015.11.001.
21. Matsui, A., Spangler, C.J., Elferich, J., Shiozaki, M., Jean, N., Zhao, X., Qin, M., Zhong, H., Yu, Z., and Gouaux, E. (2024). Cryo-electron tomographic investigation of native hippocampal glutamatergic synapses. Preprint at bioRxiv. https://doi.org/10.1101/2024.04.01.587595.
22. Schiøtz, O.H., Kaiser, C.J.O., Klumpe, S., Morado, D.R., Poege, M., Schneider, J., Beck, F., Klebl, D.P., Thompson, C., and Plitzko, J.M. (2024). Serial Lift-Out: sampling the molecular anatomy of whole organisms. Nat. Methods *21*, 1684–1692. https://doi.org/10.1038/s41592-023-02113-5.
23. Schultz, S.R., and Rolls, E.T. (1999). Analysis of information transmission in the schaffer collaterals. Hippocampus (New York, N. Y.) *9*, 582–598. https://doi.org/10.1002/(SICI)1098-1063(1999)9:5<582::AID-HIPO12>3.0.CO.

24. Tang, G., Peng, L., Baldwin, P.R., Mann, D.S., Jiang, W., Rees, I., and Ludtke, S.J. (2007). EMAN2: An extensible image processing suite for electron microscopy. J. Struct. Biol. *157*, 38–46. https://doi.org/10.1016/j.jsb.2006.05.009.

25. Korogod, N., Petersen, C.C.H., and Knott, G.W. (2015). Ultrastructural analysis of adult mouse neocortex comparing aldehyde perfusion with cryo fixation. eLife *4*, e05793. https://doi.org/10.7554/eLife.05793.

26. Shepherd, G.M., and Harris, K.M. (1998). Three-Dimensional Structure and Composition of CA3→CA1 Axons in Rat Hippocampal Slices: Implications for Presynaptic Connectivity and Compartmentalization. J. Neurosci. *18*, 8300–8310. https://doi.org/10.1523/JNEUROSCI.18-20-08300.1998.

27. Wolf, S.G., Mutsafi, Y., Dadosh, T., Ilani, T., Lansky, Z., Horowitz, B., Rubin, S., Elbaum, M., and Fass, D. (2017). 3D visualization of mitochondrial solid-phase calcium stores in whole cells. eLife *6*, e29929. https://doi.org/10.7554/eLife.29929.

28. Tao, C.-L., Liu, Y.-T., Sun, R., Zhang, B., Qi, L., Shivakoti, S., Tian, C.-L., Zhang, P., Lau, P.-M., Zhou, Z.H., and Bi, G.Q. (2018). Differentiation and Characterization of Excitatory and Inhibitory Synapses by Cryo-electron Tomography and Correlative Microscopy. J. Neurosci. *38*, 1493–1510. https://doi.org/10.1523/JNEUROSCI.1548-17.2017.

29. Du, K., Hu, L., Wang, P., and Xue, Y. (2022). Rapid isolation and cryo-EM characterization of synaptic vesicles from mammalian brain. FEBS Open Bio *12*, 1980–1987. https://doi.org/10.1002/2211-5463.13475.

30. Held, R.G., Liang, J., and Brunger, A.T. (2024). Nanoscale architecture of synaptic vesicles and scaffolding complexes revealed by cryo-electron tomography. Proc. Natl. Acad. Sci. *121*, e2403136121. https://doi.org/10.1073/pnas.2403136121.

31. Qu, L., Akbergenova, Y., Hu, Y., and Schikorski, T. (2009). Synapse-to-synapse variation in mean synaptic vesicle size and its relationship with synaptic morphology and function. J. Comp. Neurol. *514*, 343–352. https://doi.org/10.1002/cne.22007.

32. Quade, B., Camacho, M., Zhao, X., Orlando, M., Trimbuch, T., Xu, J., Li, W., Nicastro, D., Rosenmund, C., and Rizo, J. (2019). Membrane bridging by Munc13-1 is crucial for neurotransmitter release. eLife *8*, e42806. https://doi.org/10.7554/eLife.42806.

33. Imig, C., López-Murcia, F.J., Maus, L., García-Plaza, I.H., Mortensen, L.S., Schwark, M., Schwarze, V., Angibaud, J., Nägerl, U.V., Taschenberger, H., et al. (2020). Ultrastructural Imaging of Activity-Dependent Synaptic Membrane-Trafficking Events in Cultured Brain Slices. Neuron *108*, 843–860. https://doi.org/10.1016/j.neuron.2020.09.004.

34. Croft, C.L., Futch, H.S., Moore, B.D., and Golde, T.E. (2019). Organotypic brain slice cultures to model neurodegenerative proteinopathies. Mol. Neurodegener. *14*, 45. https://doi.org/10.1186/s13024-019-0346-0.

35. Peukes, J., Lovatt, C., Leistner, C., Boulanger, J., Morado, D.R., Fuller, M., Kukulski, W., Zhu, F., Komiyama, N.H., Briggs, J., et al. (2024). The molecular infrastructure of glutamatergic synapses in the mammalian forebrain. eLife *13*, RP100335. https://doi.org/10.7554/eLife.100335.1.

36. Zuber, B., Nikonenko, I., Klauser, P., Muller, D., and Dubochet, J. (2005). The mammalian central nervous synaptic cleft contains a high density of periodically organized complexes. Proc. Natl. Acad. Sci. *102*, 19192–19197. https://doi.org/10.1073/pnas.0509527102.

37. Sartori, N., Richter, K., and Dubochet, J. (1993). Vitrification depth can be increased more than 10-fold by high-pressure freezing. J. Microsc. *172*, 55–61. https://doi.org/10.1111/j.1365-2818.1993.tb03393.x.

38. Zens, B., Fäßler, F., Hansen, J.M., Hauschild, R., Datler, J., Hodirnau, V.-V., Zheden, V., Alanko, J., Sixt, M., and Schur, F.K.M. (2024). Lift-out cryo-FIBSEM and cryo-ET reveal the ultrastructural landscape of extracellular matrix. J. Cell Biol. *223*, e202309125. https://doi.org/10.1083/jcb.202309125.

39. Xue, W., Li, H., Xu, J., Yu, X., Liu, L., Liu, H., Zhao, R., and Shao, Z. (2024). Effective cryopreservation of human brain tissue and neural organoids. Cell Rep. Methods *4*, 100777. https://doi.org/10.1016/j.crmeth.2024.100777.

40. Ting, J.T., Lee, B.R., Chong, P., Soler-Llavina, G., Cobbs, C., Koch, C., Zeng, H., and Lein, E. (2018). Preparation of Acute Brain Slices Using an Optimized N-Methyl-D-glucamine Protective Recovery Method. J. Vis. Exp. *53825*, 53825. https://doi.org/10.3791/53825.

41. Ning, J., Glausier, J.R., Hsieh, C., Schmelzer, T., Buck, S.A., Franks, J., Hampton, C.M., Lewis, D.A., Marko, M., and Freyberg, Z. (2023). Cryo-FIB workflow for imaging brain tissue via in situ cryo-electron microscopy. Preprint at bioRxiv. https://doi.org/10.1101/2023.02.11.528064.

42. Leistner, C., Wilkinson, M., Burgess, A., Lovatt, M., Goodbody, S., Xu, Y., Deuchars, S., Radford, S.E., Ranson, N.A., and Frank, R.A.W. (2023). The in-tissue molecular architecture of β-amyloid pathology in the mammalian brain. Nat. Commun. *14*, 2833. https://doi.org/10.1038/s41467-023-38495-5.

43. Zhang, J., Zhang, D., Sun, L., Ji, G., Huang, X., Niu, T., Xu, J., Ma, C., Zhu, Y., Gao, N., et al. (2021). VHUT-cryo-FIB, a method to fabricate frozen hydrated lamellae from tissue specimens for *in situ* cryo-electron tomography. J. Struct. Biol. *213*, 107763. https://doi.org/10.1016/j.jsb.2021.107763.

44. Gilbert, M.A.G., Fatima, N., Jenkins, J., O'Sullivan, T.J., Schertel, A., Halfon, Y., Wilkinson, M., Morrema, T.H.J., Geibel, M., Read, R.J., et al. (2024). CryoET of β-amyloid and tau within postmortem Alzheimer's disease brain. Nature *631*, 913–919. https://doi.org/10.1038/s41586-024-07680-x.

45. Berger, C., Watson, H., Naismith, J., Dumoux, M., and Grange, M. (2024). Xenon plasma focused ion beam lamella fabrication on high-pressure frozen specimens for structural cell biology. Preprint at bioRxiv. https://doi.org/10.1101/2024.06.20.599830.

46. Szirmai, I., Buzsáki, G., and Kamondi, A. (2012). 120 years of hippocampal Schaffer collaterals. Hippocampus (New York, N. Y.) *22*, 1508–1516. https://doi.org/10.1002/hipo.22001.

47. Nicholson, D.A., Trana, R., Katz, Y., Kath, W.L., Spruston, N., and Geinisman, Y. (2006). Distance-Dependent Differences in Synapse Number and AMPA Receptor Expression in Hippocampal CA1 Pyramidal Neurons. Neuron *50*, 431–442. https://doi.org/10.1016/j.neuron.2006.03.022.

48. Lamm, L., Zufferey, S., Righetto, R.D., Wietrzynski, W., Yamauchi, K.A., Burt, A., Liu, Y., Zhang, H., Martinez-Sanchez, A., Ziegler, S., et al. (2024). MemBrain v2: an end-to-end tool for the analysis of membranes in cryo-electron tomography. Preprint at bioRxiv. https://doi.org/10.1101/2024.01.05.574336.

49. Schindelin, J., Arganda-Carreras, I., Frise, E., Kaynig, V., Longair, M., Pietzsch, T., Preibisch, S., Rueden, C., Saalfeld, S., Schmid, B., et al. (2012). Fiji: an open-source platform for biological-image analysis. Nat. Methods *9*, 676–682. https://doi.org/10.1038/nmeth.2019.

50. Kremer, J.R., Mastronarde, D.N., and McIntosh, J.R. (1996). Computer Visualization of Three-Dimensional Image Data Using IMOD. J. Struct. Biol. *116*, 71–76. https://doi.org/10.1006/jsbi.1996.0013.

51. Tegunov, D., and Cramer, P. (2019). Real-time cryo-electron microscopy data preprocessing with Warp. Nat. Methods *16*, 1146–1152. https://doi.org/10.1038/s41592-019-0580-y.

52. Pettersen, E.F., Goddard, T.D., Huang, C.C., Couch, G.S., Greenblatt, D. M., Meng, E.C., and Ferrin, T.E. (2004). UCSF Chimera—A visualization system for exploratory research and analysis. J. Comput. Chem. *25*, 1605–1612. https://doi.org/10.1002/jcc.20084.

53. Goddard, T.D., Huang, C.C., Meng, E.C., Pettersen, E.F., Couch, G.S., Morris, J.H., and Ferrin, T.E. (2018). UCSF ChimeraX: Meeting modern challenges in visualization and analysis. Protein Sci. *27*, 14–25. https://doi.org/10.1002/pro.3235.

54. Lamm, L., Righetto, R.D., Wietrzynski, W., Pöge, M., Martinez-Sanchez, A., Peng, T., and Engel, B.D. (2022). MemBrain: A deep learning-aided pipeline for detection of membrane proteins in Cryo-electron tomograms. Comput. Methods Programs Biomed. *224*, 106990. https://doi.org/10.1016/j.cmpb.2022.106990.

55. Liu, Y.-T., Zhang, H., Wang, H., Tao, C.-L., Bi, G.-Q., and Zhou, Z.H. (2022). Isotropic reconstruction for electron tomography with deep learning. Nat. Commun. *13*, 6482. https://doi.org/10.1038/s41467-022-33957-8.

56. Zheng, S., Wolff, G., Greenan, G., Chen, Z., Faas, F.G.A., Bárcena, M., Koster, A.J., Cheng, Y., and Agard, D.A. (2022). AreTomo: An integrated software package for automated marker-free, motion-corrected cryo-electron tomographic alignment and reconstruction. J. Struct. Biol. X *6*, 100068. https://doi.org/10.1016/j.yjsbx.2022.100068.

57. King, O.N. f, Bellos, D., and Basham, M. (2022). Volume Segmantics: A Python Package for Semantic Segmentation of Volumetric Data Using Pre-trained PyTorch Deep Learning Models. J. Open Source Softw. *7*, 4691. https://doi.org/10.21105/joss.04691.

58. Van Hoeymissen, E., Philippaert, K., Vennekens, R., Vriens, J., and Held, K. (2020). Horizontal Hippocampal Slices of the Mouse Brain. J. Vis. Exp. *163*, e61753. https://doi.org/10.3791/61753.

59. Papouin, T., and Haydon, P.G. (2018). Obtaining Acute Brain Slices. Bio. Protoc. *8*, e2699. https://doi.org/10.21769/BioProtoc.2699.

60. Croft, C.L., and Noble, W. (2018). Preparation of organotypic brain slice cultures for the study of Alzheimer's disease. F1000Res. *7*, 592. https://doi.org/10.12688/f1000research.14500.2.

61. Sofroniew, N., Lambert, T., Bokota, G., Nunez-Iglesias, J., Sobolewski, P., Sweet, A., Gaifas, L., Evans, K., Burt, A., Doncila Pop, D., et al. (2024). Napari: A Multi-Dimensional Image Viewer for Python (Zenodo). https://doi.org/10.5281/zenodo.13850834 Version v0.5.4rc1. .

62. Harris, C.R., Millman, K.J., van der Walt, S.J., Gommers, R., Virtanen, P., Cournapeau, D., Wieser, E., Taylor, J., Berg, S., Smith, N.J., et al. (2020). Array programming with NumPy. Nature *585*, 357–362. https://doi.org/10.1038/s41586-020-2649-2.

63. Virtanen, P., Gommers, R., Oliphant, T.E., Haberland, M., Reddy, T., Cournapeau, D., Burovski, E., Peterson, P., Weckesser, W., Bright, J., et al. (2020). SciPy 1.0: fundamental algorithms for scientific computing in Python. Nat. Methods *17*, 261–272. https://doi.org/10.1038/s41592-019-0686-2.

64. van der Walt, S., Schönberger, J.L., Nunez-Iglesias, J., Boulogne, F., Warner, J.D., Yager, N., Gouillart, E., and Yu, T.; scikit-image contributors (2014). scikit-image: image processing in Python. PeerJ *2*, e453. https://doi.org/10.7717/peerj.453.

65. Danita, C., Chiu, W., and Galaz-Montoya, J.G. (2022). Efficient manual annotation of cryogenic electron tomograms using IMOD. STAR Protoc. *3*, 101658. https://doi.org/10.1016/j.xpro.2022.101658.

66. Wagner, T., Merino, F., Stabrin, M., Moriya, T., Antoni, C., Apelbaum, A., Hagel, P., Sitsel, O., Raisch, T., Prumbaum, D., et al. (2019). SPHIRE-crYOLO is a fast and accurate fully automated particle picker for cryo-EM. Commun. Biol. *2*, 218–313. https://doi.org/10.1038/s42003-019-0437-z.

67. Grange, M., Vasishtan, D., and Grünewald, K. (2017). Cellular electron cryo tomography and *in situ* sub-volume averaging reveal the context of microtubule-based processes. J. Struct. Biol. *197*, 181–190. https://doi.org/10.1016/j.jsb.2016.06.024.

STAR★METHODS

KEY RESOURCES TABLE

REAGENT or RESOURCE	SOURCE	IDENTIFIER
Chemicals, peptides, and recombinant proteins		
Hoechst 33342	Invitrogen	Cat#11544876
Dextran 40 (MW 35k-45k)	Biosynth	Cat#YD01481
Sucrose	Sigma-Aldrich	Cat# S9378
Experimental models: Organisms/strains		
Mouse: C57BL/6J	The Jackson Labs	RRID:IMSR_JAX:000664
Software and algorithms		
Fiji	Schindelin et al.[49]	https://imagej.net/software/fiji/
IMOD	Kremer et al.[50]	https://bio3d.colorado.edu/imod/
Warp 1.0.9	Tegunov and Cramer[51]	https://github.com/warpem/warp
Amira 2021.1	Thermo Fisher Scientific	https://www.thermofisher.com/uk/en/home/electron-microscopy/products/software-em-3d-vis/amira-software/cell-biology.html
Chimera	Pettersen et al.[52]	https://www.cgl.ucsf.edu/chimera/
ChimeraX	Goddard et al.[53]	https://www.cgl.ucsf.edu/chimerax/
Membrain v2	Lamm et al.[48,54]	https://github.com/CellArchLab/MemBrain
Tomography 5	Thermo Fisher Scientific	https://www.thermofisher.com/uk/en/home/electron-microscopy/products/software-em-3d-vis/tomography-software.html
Maps 3	Thermo Fisher Scientific	https://www.thermofisher.com/uk/en/home/electron-microscopy/products/software-em-3d-vis/maps-software.html
AutoTEM Cryo	Thermo Fisher Scientific	https://www.thermofisher.com/uk/en/home/electron-microscopy/products/software-em-3d-vis/autotem-5-software.html
EMAN2	Tang et al.[24]	https://blake.bcm.edu/emanwiki/EMAN2
Isonet	Liu et al.[55]	https://isonetcryoet.com/
Python 3.9	N/A	https://www.python.org/
AreTomo	Zheng et al.[56]	msg.ucsf.edu/software
Other		
Leica VT 1200S Vibratome	Leica Microsystems	https://www.leicabiosystems.com/en-gb/research/vibratomes/leica-vt1200-s/
Leica EM Ice High Pressure Freezer	Leica Microsystems	https://www.leica-microsystems.com/products/sample-preparation-for-electron-microscopy/p/leica-em-ice/
Stellaris 8 Cryo Confocal microscope	Leica Microsystems	https://www.leica-microsystems.com/products/confocal-microscopes/p/stellaris-cryo/
Helios “G5” Hydra CX Plasma FIB/SEM	Thermo Fisher Scientific	https://www.thermofisher.com/uk/en/home/electron-microscopy/products/dualbeam-fib-sem-microscopes/helios-hydra-dualbeam.html
Delmic METEOR	Delmic	https://www.delmic.com/en/products/cryo-solutions/meteor

(*Continued on next page*)

CellPress
OPEN ACCESS

Continued

REAGENT or RESOURCE	SOURCE	IDENTIFIER
Arctis Plasma FIB/SEM	Thermo Fisher Scientific	https://www.thermofisher.com/uk/en/home/electron-microscopy/products/dualbeam-fib-sem-microscopes/arctis-cryo-pfib.html?cid=fl-arctis
Titan Krios G4 Cryo-TEM with Selectris Energy Filter and Falcon 4i Detector	Thermo Fisher Scientific	https://www.thermofisher.com/uk/en/home/electron-microscopy/products/transmission-electron-microscopes/krios-cryo-tem.html
Vesicle measurement code	This paper	https://github.com/rosalindfranklininstitute/vesicle_measurement
Model trained for Volume Segmantics[57]	This paper	https://doi.org/10.5281/zenodo.15322367

EXPERIMENTAL MODEL AND STUDY PARTICIPANT DETAILS

Animal handling and brain dissection

All animal experiments were monitored and facilitated by Named Animal Care and Welfare Officers from the Mary Lyon Center for Mouse Genetics at MRC Harwell, and animals were treated in accordance with the UK Animal Scientific Procedures Act (1986). C57BL/6J mice (Jackson Labs, Bar Harbor, ME) were provided by the Mary Lyon Center for all datasets collected.

Adult mice were euthanised in a schedule 1 procedure via overdose with an intraperitoneal injection of dilute pentobarbital in buffered saline solution (1:1). Death was confirmed through permanent cessation of circulation via cutting of the femoral artery. In total 11 mice were used in this study. Acute hippocampal slices for cryoET evaluation of the CA1 architecture were prepared from one male and one female 4–6-month-old C57BL/6J mouse. For the cortex experiment datasets, a 5-month-old female C57BL/6J mouse was used. When assessing vitrification and optimising early lift-out methodologies, samples from mice aged 7 to 184-days-old (male and female) were high-pressure frozen. Mouse pups (between ages of 7–21-days-old) were euthanised as described previously but instead using an intraperitoneal injection of 200 mg mL^{-1} concentrated pentobarbital.

METHOD DETAILS

Sectioning of acute brain slices

The slice preparation protocol was modified from established protocols.[58–60] In brief, following brain dissection, the cerebellum was removed and the hemispheres separated at the longitudinal fissure using a scalpel. The remaining tissue was glued cut face down onto a chuck, and 100, 150, or 200 μm sagittal slices were made using a Leica VT 1200S Vibratome set at a slicing speed of 0.7 mm s^{-1} and an amplitude of 1 mm in ice-cold dissection medium (99% HBSS, 0.035% w/v ascorbic acid, 0.002% w/v ATP, 1% v/v 100x penicillin/streptomycin solution, filter-sterilised through a 0.22 μm membrane, pH 7.1). Two brains were sectioned while bubbling with carbogen (95% O_2/5% CO_2): these resulted in slices of cortex (where data are presented) and datasets from the 172-day-old female mouse ("CA1-sr 2" and "CA1-sr 4"). Where cortex was used, biopsy punches were taken directly from sagittal sections. For hippocampus biopsies, each hippocampus was first isolated using curved tip teasing needles (Bochem) and individual hippocampi transferred to a well of a 24-well plate with resting media (300 μL Hibernate-A medium (Gibco) supplemented with 2% v/v B-27 (Gibco)) for recovery and transport and kept on ice until high-pressure frozen.

High-pressure freezing

3 mm type B gold-plated copper high-pressure freezing carriers (Leica Microsystems) were prepared as previously described.[14] Briefly, the flat side was sanded with 4000 grit sandpaper to remove machining marks followed by 10,000 grit sandpaper to remove harsh aberrations followed by metal polish to smooth the surface. Sanded and polished type B carriers and type A carriers were incubated in hexadecene (Sigma-Aldrich) for at least 45 min prior to use. Type A carriers containing tissue were pen-marked on the rim to aid in determining carrier orientation for subsequent imaging and serial lift-out.

Tissue slices in resting media were incubated with Hoechst 33342 (Invitrogen) nuclear stain for 5 min prior to target excision with a 2 mm biopsy punch. Biopsies were transferred via transfer pipette to a 24-well plate with cryoprotectant in buffer (Table S2) where they were allowed to incubate at room temperature for the specified time ranging from 0 to 30 min. After incubation, tissue biopsies were transferred to the 0.1 mm (100 μm thick tissue sections) or 0.2 mm (150 or 200 μm thick tissue sections) recessed side of 3 mm type A gold-plated copper HPF carriers (Leica Microsystems).

In total, brain dissection, vibratome slicing, and hippocampal isolation from all slices took approximately 1 h, followed by approximately 20 min of slice incubation with cryoprotectant to allow for complete vitrification. Each slice took several minutes to manipulate

and high-pressure freeze, and only slices which appeared optimal in both fluorescence and the FIB/SEM were used for subsequent lift-out and data collection. This resulted in postmortem intervals for slices used for data collection ranging from 1.5–4 h.

Cryo-confocal microscopy

After freezing, carriers were screened on a Stellaris 8 Cryo Confocal Microscope (Leica Microsystems) fitted with a cryostage. Imaging in fluorescence and reflection modes was performed in camera mode with an HC PL APO 50 ×0.9-NA objective using the LAS X software (Leica Microsystems). Tilesets were acquired with 20% overlap between tiles and merged with maximum intensity projections calculated in the LAS X software for fluorescence and reflection modes. An outline of the tissue section was often visible in reflection mode and used in combination with the carrier rim to aid in alignment between reflection and fluorescence channels. The mark drawn onto the carrier rim was visible in reflection mode and used for orientation-conscious carrier loading into the FIB/SEM.

Hippocampal layer targeting

Carriers were oriented for loading into the Helios "G5" Hydra CX plasma FIB/SEM (Thermo Fisher Scientific) using previously obtained orientation information from the cryo-confocal microscope. Samples were typically loaded into the 27° pre-tilted shuttle (Thermo Fisher Scientific) such that the CA1-sp would face down with the dentate gyrus facing up. This would allow the CA1-sp to be oriented up in the fluorescence module and the long trench required for perpendicular lift-out to stretch into the CA1-slm such that lift-outs could be obtained from both the CA1-sr and the directly overlaying CA1-sp. This orientation maximised the number of lift-outs that could be obtained from these layers of interest.

Five uniquely shaped fiducial markers were milled into the tissue sample surface that would be visible in the SEM and the integrated fluorescence module (IFM). These were composed of 75 μm × 4 μm × 3 μm Z depth (Si) rectangle patterns milled at 60 nA with xenon plasma. One fiducial was placed near the center of the carrier to aid in centering the carrier in the IFM for fluorescence tileset acquisition.

A Meteor fluorescence microscope (Delmic) incorporated onto the Helios Hydra FIB/SEM and equipped with a 20x (NA 0.45) objective was used to acquire tilesets that would span the entire carrier in X and Y. This was 11 × 9 tiles (1.88 mm × 1.87 mm) with 10 Z steps of 2 μm per step. Laser power was set to 500 mW and 150 ms exposures were acquired. Maximum intensity projections were calculated in the Odemis software (Delmic).

Fiducials visible in the fluorescence image were used as reference points for targeting specific hippocampal layers. If fiducials were far from the target region, smaller patterns (30 μm line with 3 μm Z-depth) were milled using 4 nA xenon plasma current adjacent to the lift-out target region and checked in the fluorescence module before trench milling to ensure fine targeting. For CA1-sr, targets were placed up to halfway between the CA1-sp and the visible CA1-slm (Figure 1). Targets were lifted out from between 50 and 150 μm from the CA1-sp pyramidal cell layer nuclei fluorescence.

Cryo-lift-out

Serial cryo-lift-out was performed on a Helios "G5" Hydra CX plasma FIB/SEM (Thermo Fisher Scientific) equipped with a tungsten EasyLift needle. A copper block of 15 μm (width) by 10 μm (depth) by 12 μm (height) was taken from the receiver grid and attached to the end of the needle by redeposition welding.[22] The overall process of lift-out, and the milling parameters used at each step for both perpendicular and planar approaches are described in Figure S1 and Table S2 respectively.

For perpendicular lift-out, a target area of 60 μm in X and 30 μm in Y was chosen based on position relative to layers observed in the fluorescence module. The stage was compucentrically rotated such that the sample would be perpendicular to the FIB (Figure S1). For our stage with a 27° pre-tilt sample shuttle, this implied a stage tilt of 25°. A long trench of 60 μm in X by 150–200 μm in Y by 5–6 μm in Z was milled with an RCS pattern behind the target region such that the long trench would be at the front of the sample after compucentric rotation with scan rotation set to 180° (Figure S1 and Table S2). A second, shorter, but deeper trench (RCS pattern) was milled in front of the sample to allow for subsequent assessment of completeness of side and undercuts in the next step. This milling pattern was 60 μm in X by 40–60 μm in Y by 6–11 μm in Z. Larger Z depths were used in instances where longer trenches were also used (Table S2). The scan direction of both trench RCS patterns were oriented toward the lift-out target area. Side and undercuts were made with the long trench facing toward the FIB at a stage rotation of 8°–15° with our 27° pre-tilted shuttle. For side cuts (15 nA), patterns were 4 μm wide with at least 45 μm between patterns. For undercuts, patterns were 6–8 μm tall (Figure S1).

For sample attachment to the needle, the stage was rotated to between −5 and 5° – or the shallowest angle where the entirety of the remaining tabs leftover from the side cuts could be seen. The tissue was then attached to the copper block adapter on the EasyLift needle by redeposition welding (Figure S1). Once attached, the remaining tabs on the tissue were milled away with 4 μm wide rectangle milling patterns at 4 nA. The EasyLift needle with attached tissue sample was then retracted.

Planar lift-out experiments were undertaken based on the schematic depicted in Figure S1. A target area of 60 μm in X and 350–400 μm in Y was chosen based on position relative to layers observed in the fluorescence module. The stage was compucentrically rotated such that the sample would be perpendicular to the FIB. Two trenches of 50 μm in X, 350–400 μm in Y and 6 μm in Z were milled either side of the region of interest using 200 nA (Figure S1). A third trench was milled at the base of the region measuring 210 μm in X, 60 μm in Y and 2 μm in Z for copper block adapter attachment (Table S2). This left the region connected to the bulk of the material. The scan direction of both trench RCS patterns were oriented toward the lift-out target area. Undercuts were performed at ±90° stage rotation relative to the region of interest, with a −15° stage tilt. Rectangular patterns that span the width of

the lift-out region (350–400 μm) in X, 10 μm in Y and 1 μm in Z were placed 10 μm below the surface of the sample. Milling was performed at 15 nA until no material connected the region and the bulk of the sample, confirmed by SEM/FIB imaging.

For sample attachment to the needle, the stage was rotated perpendicular to the FIB. The tissue was then attached to the copper block adapter on the EasyLift needle by redeposition welding (Figure S1). Once attached, the material connecting the side of the region and the bulk sample was milled away with 5 μm wide rectangle milling patterns at 4 nA. The EasyLift needle with attached tissue sample was then retracted.

Serial section deposition

Serial sections were deposited onto rectangular pattern 400 x 100 mesh TEM support grids (Agar Scientific). The base and sides of the lift-out chunk were trimmed with 4 nA to match the diameter of the receiving grid. The stage was rotated to the shallowest angle possible, here −5 to −3°, before the lift-out chunk was brought down into contact with the grid. 3–5 μm sections were then milled off using a line pattern at 1–4 nA. While 4 nA line patterns were faster (<2 min/section), 1 nA line patterns (∼5 min/section) yielded a smoother surface that would be advantageous for subsequent thinning steps, thus 1 nA was used for sectioning in most cases. Following deposition, the stage was tilted to 15° and welding patterns were placed on either side of each section for attachment to the grid by redeposition welding. These patterns were CCS oriented toward the copper bars with a 30 μs dwell time. Patterns were 3 μm long by 0.8 μm tall with approximately 4 μm periodicity. This resulted in 6–8 welds per section and took ∼30 s/weld. After all sections were deposited and welded, GIS was applied for 70 s to achieve a few hundred nm thick GIS layer.

Fine milling of serial lift-out sections

Thinning of serial sections was carried out on an Arctis plasma FIB (Thermo Fisher Scientific) initially using AutoTEM Cryo (Thermo Fisher Scientific) for automated thinning with xenon plasma down to approximately 400–600 nm thickness depending on section quality where higher quality sections could be automatically thinned to lower values. Lamella width was set to 15–18 μm with a target thickness of 110 nm and a Z depth of 2.5 μm in silicon. For rough milling, rectangle patterns with a beam current of 4 nA with 2–3 μm pattern offsets were used to bring the total lamella thickness down to∼4–6 μm. For medium milling, a cleaning cross section was used with 0.7–1.0 μm offsets with a beam current of 1 nA to bring the lamella thickness down to∼1.4–2 μm. For fine milling, cleaning cross section patterns with 120–300 nm offsets were placed and a beam current of 0.1 nA was used to mill sections down 400–600 nm before switching to manual polishing steps with argon plasma to bring the final thickness down to ∼150–300 nm using 60 pA and 20 pA beam currents. For all automated milling steps, we found that the lower end of the thickness spectrum given at each step could be used for high quality, smooth, stable starting sections. Lower quality sections with rough surfaces required switching to manual polishing at greater (∼600nm) thicknesses to avoid breakage and further curtain propagation with xenon plasma.

Cryo-electron tomography tilt series acquisition

TEM image acquisition was carried out on a Titan Krios G4 (Thermo Fisher Scientific) electron microscope operating at 300 kV and equipped with a ±90° stage, a Selectris Energy Filter (Thermo Fisher Scientific), and a Falcon 4i direct electron detector camera (Thermo Fisher Scientific). For all acquisitions, an energy selecting slit width of 10 eV was used. Dose-symmetric tilt series were collected utilising an image-shift/beam-shift data collection strategy in Tomo5 version 5.17.0.6390 (Thermo Fisher Scientific). Tilt series were acquired as movies using the.EER file format at a nominal magnification of 42,000× (3.05 Å/pixel) or 64,000x (1.98 Å/pixel) in counting mode from +60° to −60° starting from the pre-determined milling angle (i.e., zero degree offset) in 3° increments with a total dose of 130 $e^-/Å^2$ with a target defocus from −3 to −5 μm.

Tomogram reconstruction

Warp[51] version 1.0.9 was used for CTF estimation and motion correction of tilt series. Tilt series were aligned and reconstructed using AreTomo[56] version 1.3.4, filtered using EMAN2[24] and tomograms were visualized and lamella thickness measured using IMOD[50] version 4.12.56. Thickness measurements were based on selecting a position near the center of the tomogram, moving through Z until biological material was no longer visible and setting that as the starting point of the lamella. This was repeated moving through Z in the opposing direction where the distance between the two points was the measured lamella thickness. This measured thickness was then used to reconstruct tomograms again with a more accurate AlignZ parameter. Bin8 tomograms (pixel size 15.84 Å) were post-processed using Isonet.[55] Tomograms were subjected to CTF deconvolution (SNR fall-off = 0.9, deconv_strength = 0.9–1.1 depending on dataset) before missing wedge correction. All tomograms shown in figures are deconvolved, but not missing wedge corrected.

Tomogram segmentation

Membrain-seg[48,54] was used for initial segmentation of tomograms post-processed in isonet. Besides membranes, membrain-seg also segmented high contrast structures like microtubules. Membranes were further segmented using Chimera's[52] Segger tool. Microtubules and the synaptic cleft were segmented manually in Amira version 2023.1.1 (Thermo Scientific). Amira was also used to manually segment some regions of membrane and synaptic vesicles not segmented with membrain-seg and to segment vesicle tethering proteins. For manual segmentations, a 20 Å Gaussian filter was used to aid in segmentation of densities within microtubules and smoothing of membranes. Volumes were visualised in Chimera X.[53]

The package Volume Segmantics[57] was used for quantitative segmentation of synaptic vesicles. The training dataset was comprised of 22 tomograms and their corresponding binary masks, which were generated through a combination of manual annotations using Napari[61] and training intermediate models and correcting their predictions (pseudo-labelling). Different model architectures were tested to optimize the model, ultimately using a U-Net with ResNet50 encoder which was trained for a total of 13 epochs. A combination of 0.75 Binary Cross Entropy and 0.25 Dice Loss (BCEDiceLoss) was used. The segmentations were post-processed by applying a threshold for sphericity and minimum voxel size to exclude any partially segmented vesicles. Python scripts were developed to calculate key morphological metrics for each synaptic vesicle. These were: 1) diameter, 2) calculated sphere volume from the measured diameter, 3) volume calculated from a convex hull of the segmentation, and 4) sphericity based on the points in the convex hull. For each of these measures, the synaptic vesicle membrane biolayer is included and thus values represent the space taken up by the vesicles rather than the volume of their interiors. Scripts utilized the NumPy,[62] SciPy[63] and Scikit-image[64] libraries, with the volume measured by computing the convex hull of each synaptic vesicle using the ConvexHull function from the scipy.spatial module. This provided the smallest convex shape enclosing all points of each vesicle. The surface area of the convex hull was also obtained, and these values were used to calculate the sphericity of each vesicle. We note that the missing wedge will result in a smaller convex hull volume than the calculated sphere volume from the diameter and a lower calculated sphericity than may exist in reality.

Filament analysis

Microtubule backbones were traced through low-pass filtered, bin-8 tomograms to create models in 3dmod (IMOD)[50] according to a previously published protocol.[65] Briefly, for each microtubule filament observed, a point was placed every 10–20 slices through Z to generate a contour spanning the length of the filament. Model files were then converted into coordinates using the IMOD command "model2point". The coordinates were modeled as a spline and the pairwise interfilament distance was measured and visualised using custom Python scripts. The analysis of the microtubule filament frequency across the CA1 region was performed by measuring the distance from the center of each section to the top of the lift-out in FIJI.[49]

Sub-volume averaging

The microtubule backbone traces were converted to points using the method described above. The resulting points were resampled along the axis of the microtubules according to the distance of a tubulin monomer (40 Å) to generate coordinates for averaging using custom Python scripts. The coordinates were assigned initial angles facing along the filament axis using crYOLO[66] box manager toolbox "coords2priors" and the resulting STAR file was used to extract sub-volumes at 4x downsampling (pixel size = 7.92 Å) from Warp[51] with a box size of 84 pixels. Particles were then subjected to 3D refinement with helical reconstruction and symmetry applied based on 13 protofilament (pf) microtubules, using a low-pass filtered (30 Å) 13 pf structure as a template.[67] The resultant average exhibited 13 pf which was then subjected to 3D classification producing 4 classes. Three contained 13 protofilaments and a fourth contained 14 protofilaments.

QUANTIFICATION AND STATISTICAL ANALYSIS

Cellular features within tomograms were identified by eye, based on similarity of known structures previously described. The filament frequency as a function of distance and pairwise interfilament distance were measured as described in the "Filament Analysis" section of the methods. Summary statistics were calculated in Python and plotted in Figures S7B–S7D. For synaptic vesicles, segmentation was carried out as described in the "tomogram segmentation" section of the methods. Quantification of vesicle parameters were carried out as described in the legend for Figure S6 where each point represents the mean of all synaptic vesicles present in the tomogram. The "synaptic" category contained tomograms where vesicles were only present in a visible synapse defined as a pre synapse, post synapse, and synaptic cleft (27). "All" contained all vesicles in all synapse tomograms analyzed (107). For Figure S6H, the number of synapse tomograms per dataset can be found listed in Table S3. A small number of tomograms (<15) were unable to be segmented automatically due to poor data quality and were thus discarded.

Cell Reports Methods, Volume *5*

Supplemental information

A generalizable and targeted molecular biopsy approach for *in situ* cryogenic electron tomography of vitreous brain tissue

Calina Glynn, Jake L.R. Smith, Matthew Case, Rebecca Csöndör, Ana Katsini, Maria E. Sanita, Thomas S. Glen, Avery Pennington, and Michael Grange

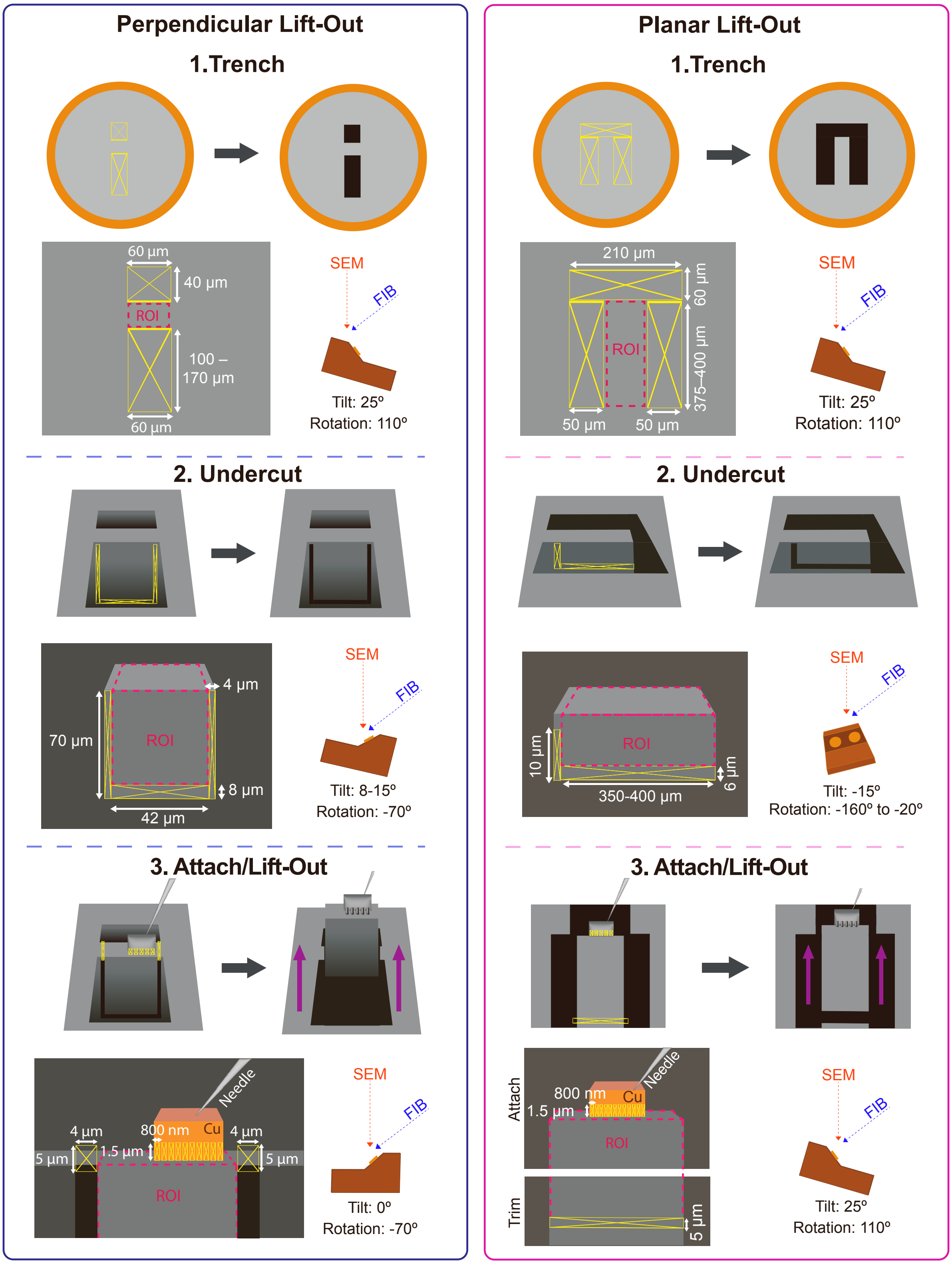
Perpendicular Lift-Out
1.Trench
60 µm
40 µm
ROI
100 – 170 µm
60 µm
SEM
FIB
Tilt: 25º
Rotation: 110º
2. Undercut
4 µm
70 µm
ROI
8 µm
42 µm
SEM
FIB
Tilt: 8-15º
Rotation: -70º
3. Attach/Lift-Out
Needle
4 µm
800 nm
Cu
4 µm
5 µm
1.5 µm
5 µm
ROI
SEM
FIB
Tilt: 0º
Rotation: -70º
Planar Lift-Out
1.Trench
210 µm
60 µm
ROI
375–400 µm
50 µm
50 µm
SEM
FIB
Tilt: 25º
Rotation: 110º
2. Undercut
10 µm
ROI
6 µm
350-400 µm
SEM
FIB
Tilt: -15º
Rotation: -160º to -20º
3. Attach/Lift-Out
Attach
800 nm
Needle
Cu
1.5 µm
ROI
Trim
5 µm
SEM
FIB
Tilt: 25º
Rotation: 110º

Figure S1. Milling Procedure for Cryo-Lift-Out from HPF Carriers. Related to Figure 1

Schematics depicting the workflow for perpendicular (left column, blue box) and planar (right column, pink box) lift-outs. Top – 1. Trench – diagrams showing the region of interest (‘ROI’, pink box), FIB milling patterns (yellow box) with measurements and stage orientations for trench milling in both perpendicular and planar samples. Middle – 2. Undercut – diagrams with FIB milling patterns (yellow box) to isolate the sample for lift out from the rest of the material for perpendicular and planar lift out. Note the difference in stage geometries for the planar lift out. Bottom – 3. Attach/Lift Out – diagram showing the redeposition FIB welding (yellow box) on the copper block (Cu) attached to the lift out needle. The purple arrow denotes the direction of lift out. Note the 180º difference in stage rotation for perpendicular versus planar lift out.

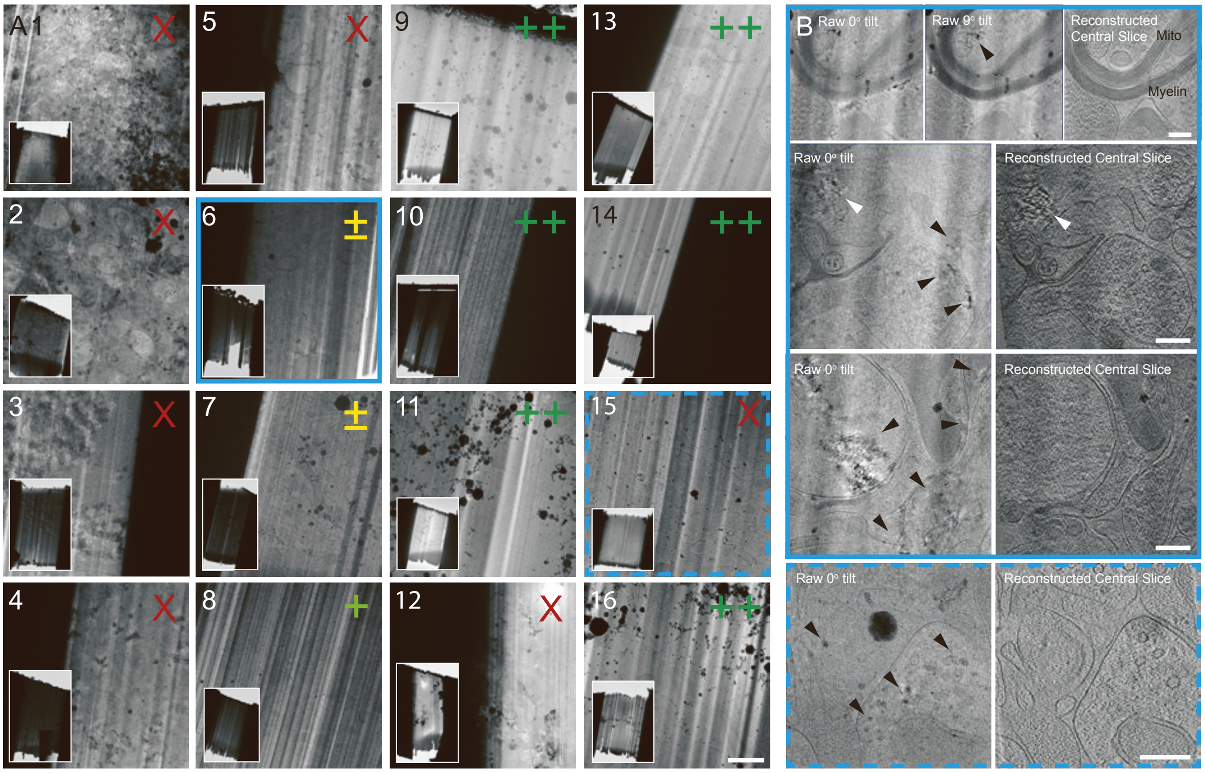

Figure S2. Vitrification Screen for Acute Mouse Brain Tissue Slices. Related to Figure 2.

(A) TEM of representative whole lamella overviews (left) and higher magnification search maps (right) from each tissue slice thickness, cryoprotectant, buffer, and incubation time combination detailed in Extended Data Table 1. "X", "±", "+" and "++" indicate degree of vitrification. An "X" represents conditions where no areas of the lamella appeared vitreous and tilt series were not able to be collected without stage tracking errors resulting from extreme fluctuations in contrast introduced by ice diffraction. A "±" indicates lamella where tilt series could be acquired, but frames in all tilt series displayed incomplete vitrification evidenced by ice diffraction. A "+" rating was given to conditions where some tilt series could be acquired without ice diffraction, but some non-vitreous ice could be seen in some tilt series. A "++" symbol was assigned to conditions where no or very few (<10%) tilt series displayed evidence of non-vitreous ice. Scale bar 2 µm.

(B) Representative raw tilts from condition 6 (top, solid outline) and condition 15 (bottom, dashed outline) illustrating that while signs of non-vitreous ice may (white arrows) or may not (black arrows) be present in tomogram reconstructions, raw tilts often displayed signed of non-vitreous ice. Scale bars: 200 nm.

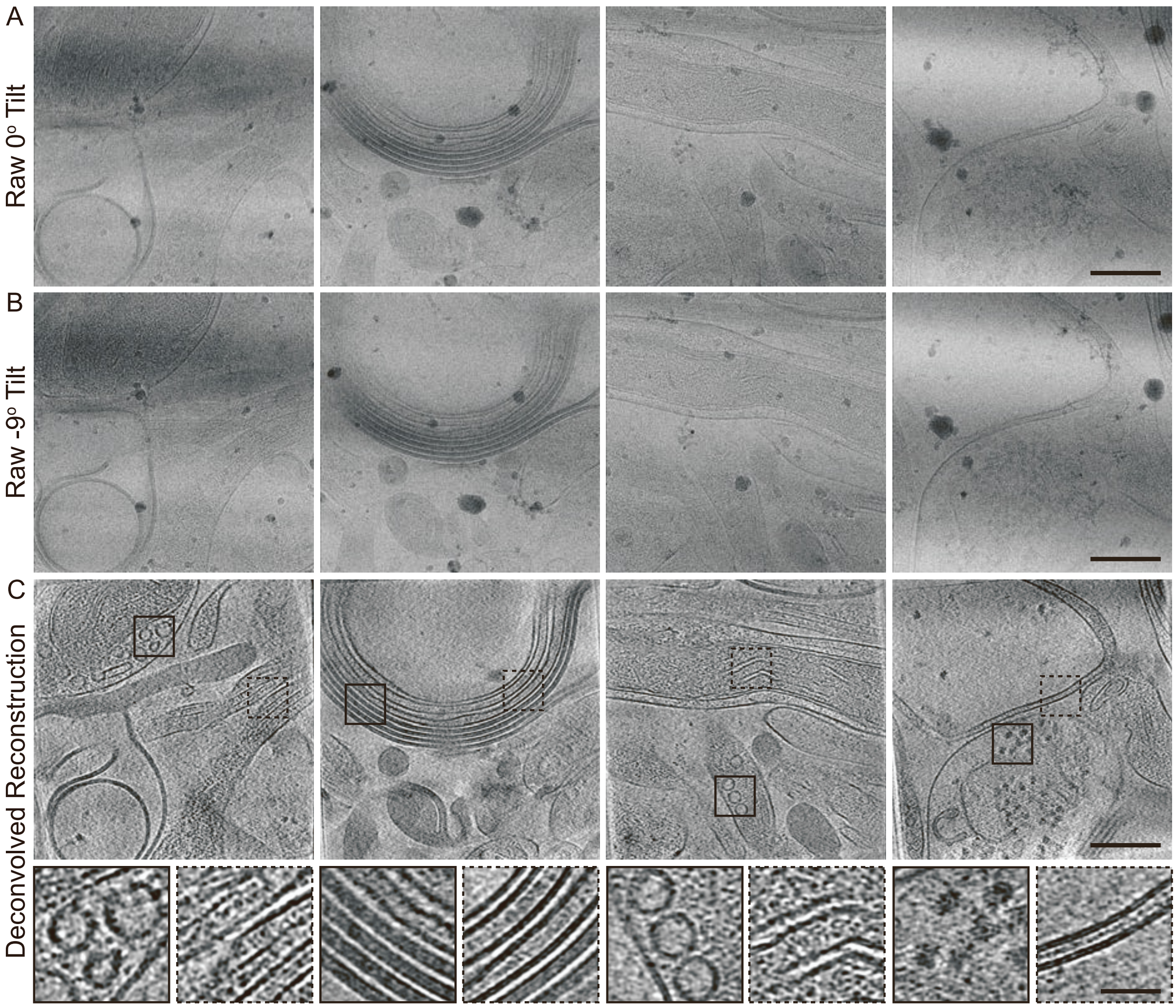

Figure S3. CryoET of Vitrified Mouse Cortex. Related to Figure 2.

(A–B) Raw tilts taken at 0 and –9º relative to the milling angle respectively, showing no diffraction from non-vitreous ice or membrane deformation indicative on a non-vitreous sample. Scale bar 200 nm

(C) Deconvolved tomogram reconstructions highlighting vesicles, microtubules, myelin, mitochondrial cristae, and ribosomes. Scale Bar in full image 200 nm, inset 50 nm

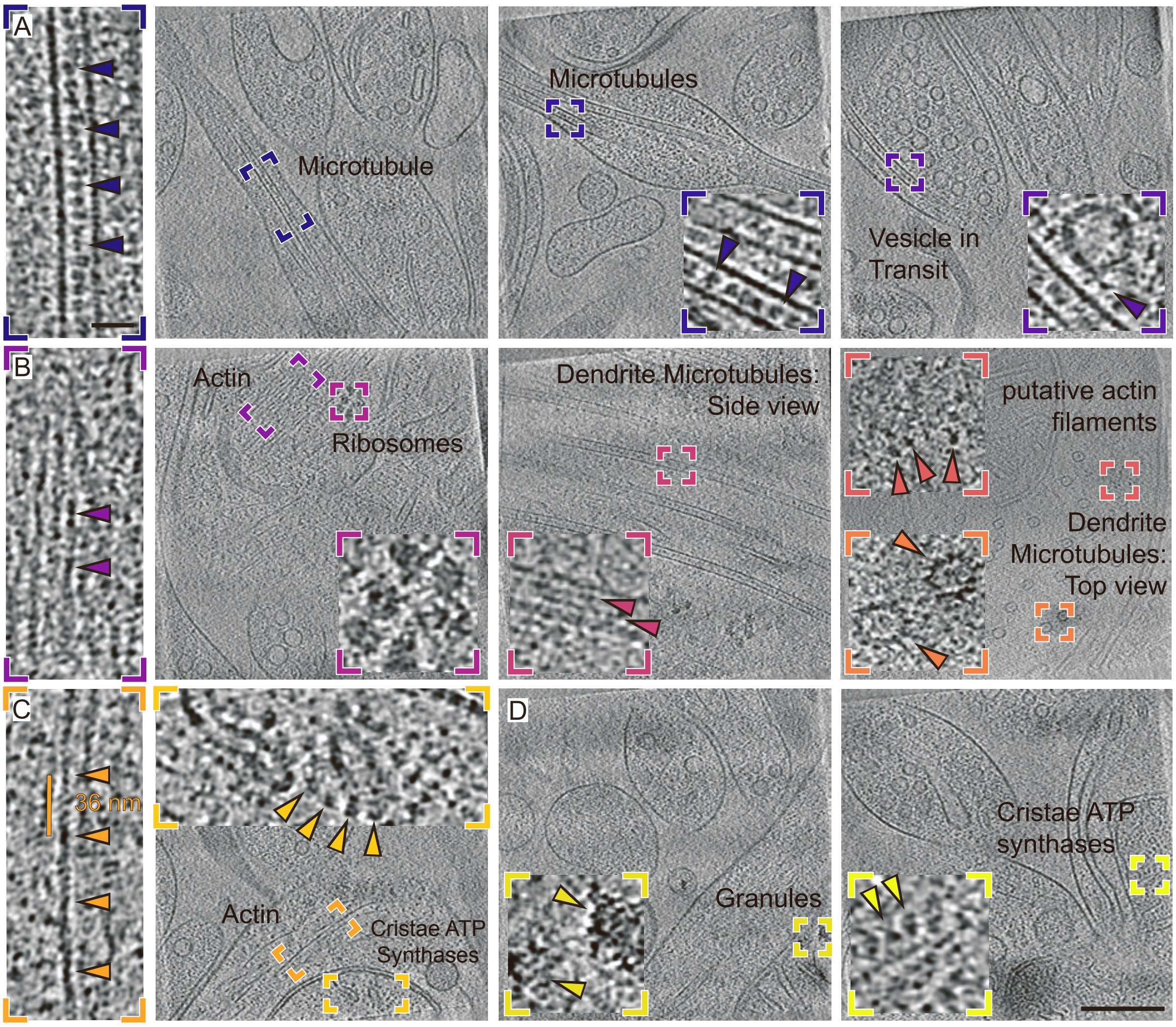

Figure S4. Features Observed in CA1-sr. Related to Figure 3.

(A) In perpendicular lift-outs from the CA1-sr we were able find abundant microtubules with luminal bodies (left and middle arrows) and in rare instances vesicles being transported along microtubules (right).

(B) We also less commonly found larger cytoskeletal organisation in the form of actin filaments (left), dendrites with microtubules traversing the entire field of view (middle, 10/359 tomograms) and very rarely head on views of dendrites with microtubule (right, bottom inset, 1/359 tomograms) and putative actin filament cross sections (right, top inset). For traversing microtubules, we could occasionally observe the lattice (arrows in middle inset) of aligned subunits.

(C) In our high-quality tomograms, actin could more clearly be identified by a 36 nm helical repeat (left inset). We were also able to independently identify cristae decorated with putative ATP synthases (top inset).

(D) We captured mitochondria with (left, 13/359 tomograms) and without (right, 119/359 tomograms) granular deposits. Putative ATPases could be observed in cristae (right inset, arrows). Scale bar for tomograms 200 nm, insets 25 nm.

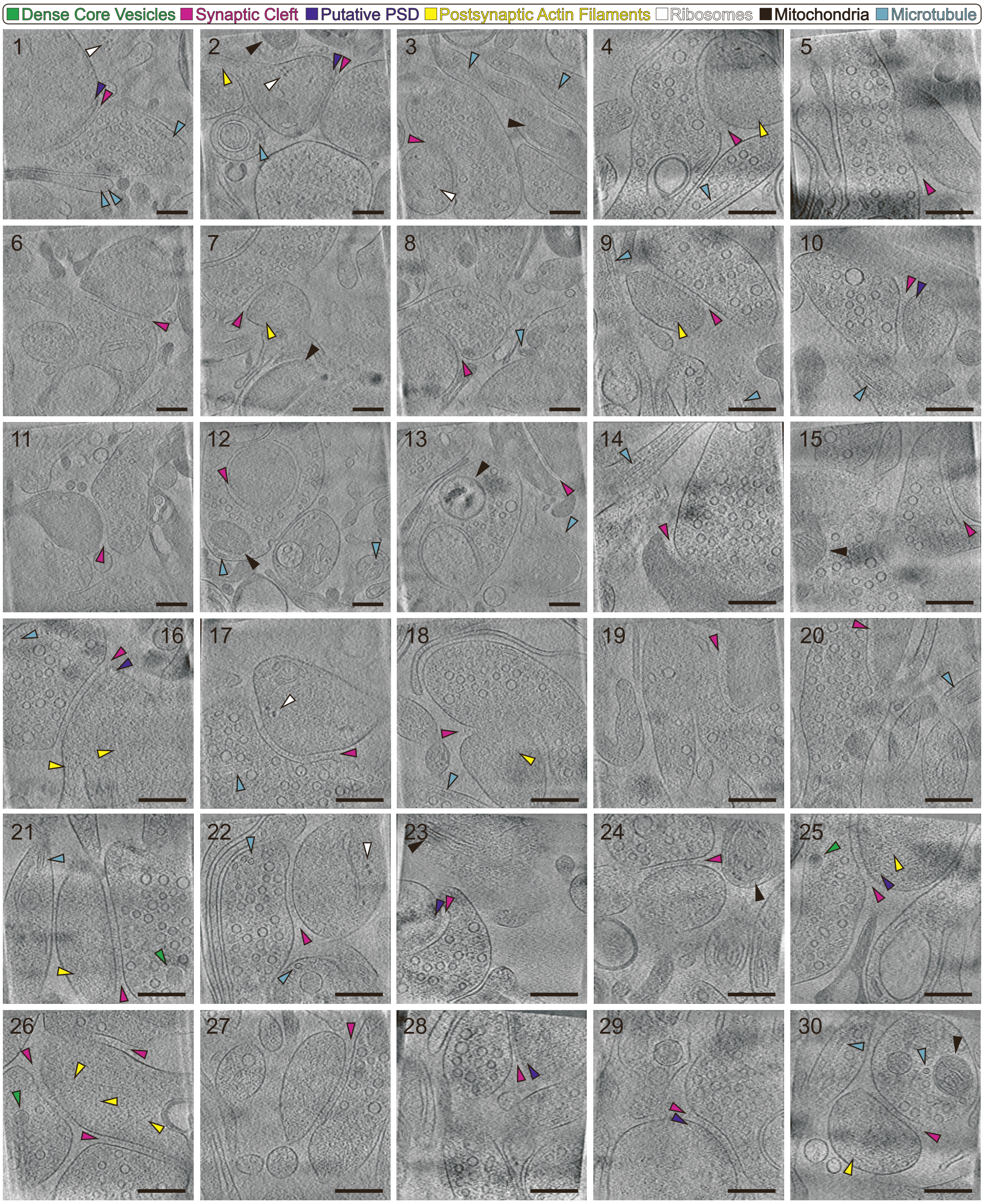

Figure S5. Synapse diversity in the CA1-sr. Related to Figures 4 and 5.

An array of 30 out of 107 total synapses representing all datasets including synapses from the planar lift-out (23–25, 28–30). Arrows highlight the synaptic cleft (pink) in all of the tomogram cross sections, dense core vesicles (blue) in the presynaptic terminal, putative postsynaptic density (PSD, dark purple) when present, postsynaptic actin filaments (light purple), ribosomes (white), mitochondria (orange) and microtubules (teal). Scale bars 200 nm.

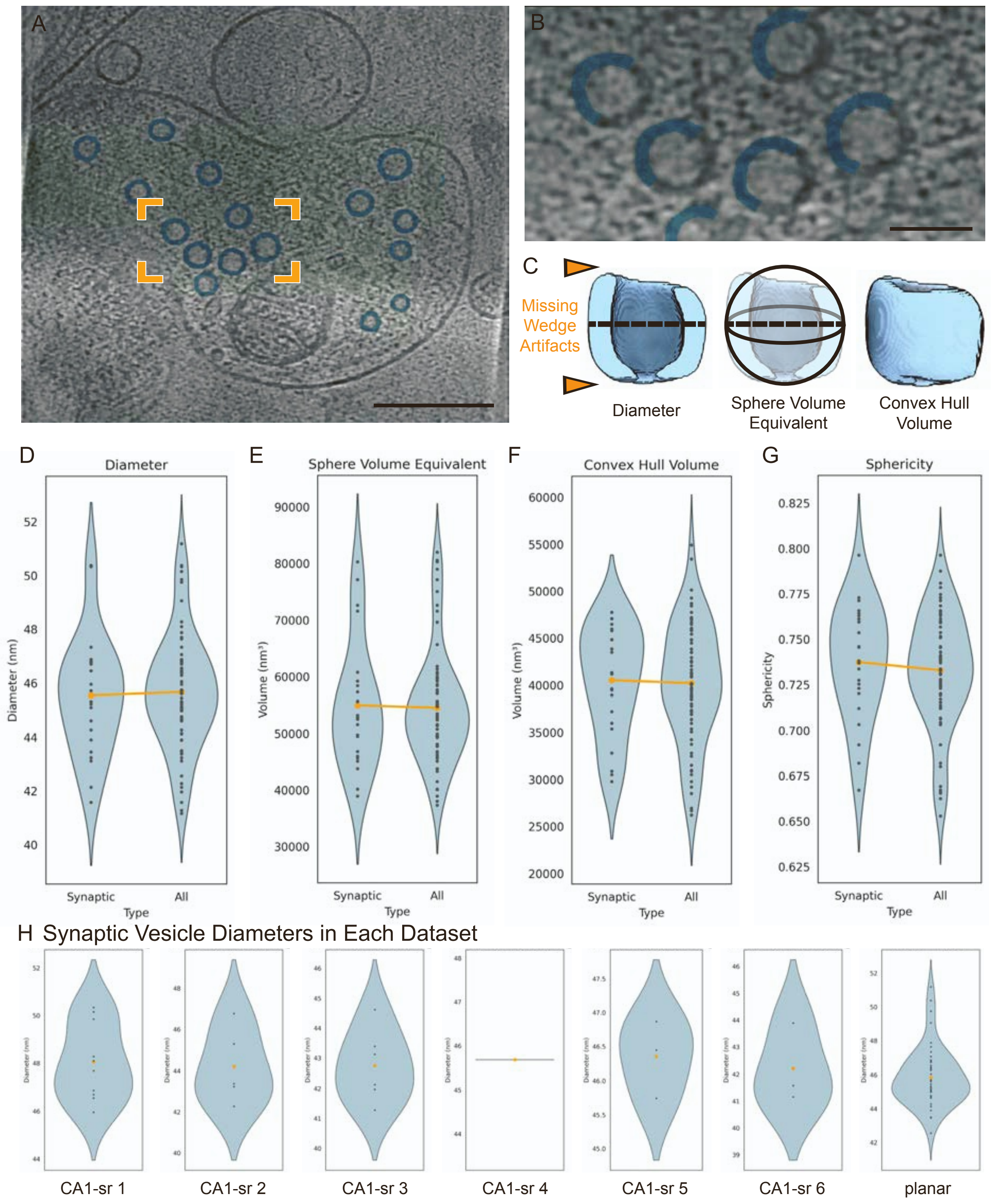

A
B
C
Missing Wedge Artifacts
Diameter
Sphere Volume Equivalent
Convex Hull Volume
D
Diameter
Diameter (nm)
Synaptic
All
Type
E
Sphere Volume Equivalent
Volume (nm³)
F
Convex Hull Volume
G
Sphericity
H Synaptic Vesicle Diameters in Each Dataset
CA1-sr 1
CA1-sr 2
CA1-sr 3
CA1-sr 4
CA1-sr 5
CA1-sr 6
planar

Figure S6. Segmentation and Size Distribution of Synaptic Vesicles. Related to Figures 4 and 5.

(A) The image shows a tomogram with 16 visible segmentations (62 vesicles in total throughout the tomogram). Scale bar: 200 nm.

(B) A crop of the tomogram with 6 vesicles, where half of the vesicle has been removed to show the degree to which the segmentation aligns with the vesicle boundaries. Scale bar: 50 nm.

(C) Illustration of the measurements used in the subsequent graphs, where diameter measures the widest diameter of the vesicle in 2D, sphere volume equivalent uses this diameter to calculate the vesicle volume assuming the vesicle is a perfect sphere, and the convex hull volume based on the volume that could encompass all points in the segmentation. All measures include the lipid bilayer.

(D) Violin plot of the mean diameter, with left plot (Synaptic) including only tomograms where all vesicles are in a visible synapse (27) and the right plot (All) includes all vesicles in all synapse tomograms (107) which could be segmented. The diameter is measured in 2D, per slice, where dots are the per-tomogram means of the median of the per-slice 2D feret diameter of each vesicle. The median was chosen to be more robust to outliers. There was no statistically significant difference between the diameters of vesicles that were part of a visible synapse and vesicles where an accompanying synapse was not captured within the tomogram.

(E) Plot of the mean Sphere Volume Equivalent per tomogram, which is the calculated volume of a sphere, given the diameter used in (D). As in (D), there was no statistically significant difference in volume between the two groups.

(F) The mean volume of the convex hull of the segmented connected component, per tomogram. The segmented component is a is the shell of the vesicle, so the convex hull is used to fill in the interior. There was no statistically significant difference found between the two groups.

(G) A violin plot of the mean sphericity of the vesicles, per tomogram. There was no statistically significant difference found between the two groups.

(H) Synaptic vesicle diameter distribution for each dataset. There was no statistically significant difference found in synaptic vesicle diameter between any of the datasets.

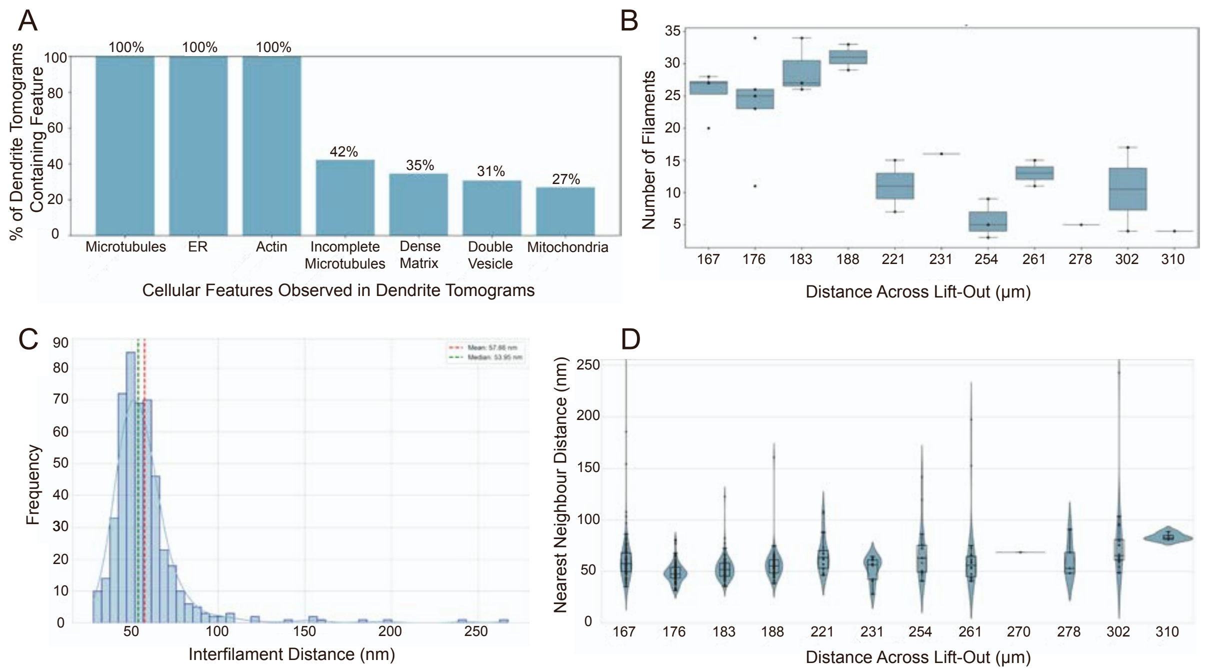

Figure S7. Analysis of the cytoskeletal composition and organization of the apical dendrite network. Related to Figures 6 and 7.

(A) Characterization of the percentage of the cellular components observed in 30 reconstructed tomograms depicting axial views of dendrites.

(B) The number of microtubules observed in dendrite tomograms as a function of distance from the CA1-so.

(C) Histogram of the nearest neighbour differences between microtubules from all 30 dendrite tomograms. Mean and median values are plotted as red and green dashed lines respectively.

(D) Violin plot of nearest neighbour distances between microtubules as a function of distance from the CA1-so.

Table S1. Conditions assessed for vitrification state of high-pressure frozen tissue. Related to Star★Methods sections: Experimental Model and Subject Details – Animal Handling and Brain Dissection and Method Details – High Pressure Freezing.

Mouse Age (days)	Brain Region	Section Thickness (μm)	Carrier Material	Carrier Recess Depth (μm)	Cryoprotectant and Buffer	Incubation Time (min)	Vitreous Rating[$]	Figure S2 Number
14	Cortex	200	Aluminium	200	20% Dextran+ in PBS pH 7.4	0	X	1
13	Cortex	150	Aluminium	150	20% Dextran in PBS pH 7.4	30	X	2
7	Cortex	150	Copper	200	20% Dextran in PBS pH 7.4	30	X	3
118	Cortex	150	Copper	200	20% BSA in 100 mM PB pH 7.4	35	X	4
118	Cortex	100	Copper	100	20% BSA in 100 mM PB pH 7.4	0	X	5
9	Cortex	150	Copper	200	10% Dextran 5% Sucrose in 100 mM PB pH 7.4	25	X	
38	Cortex	100	Copper	100	10% Dextran 5% Sucrose in 100 mM PB pH 7.4	20	+*	
125	Cortex	100	Copper	100	10% Dextran 5% Sucrose in 100 mM PB pH 7.4	25	±	6
125	Cortex	100	Copper	100	10% Dextran 5% Sucrose in 100 mM PB pH 7.4	30	±	7
125	Cortex	100	Copper	100	10% Dextran 5% Sucrose 5% Ethylene Glycol in 100 mM PB pH 7.4	20	+	8
149	Cortex	100	Copper	100	10% Dextran 5% Sucrose 5% Ethylene Glycol in 100 mM PB pH 7.4	25	++	9
125	Cortex	100	Copper	100	10% Dextran 10% Sucrose in 100 mM PB pH 7.4	20	++	10
149	Cortex	100	Copper	100	10% Dextran 10% Sucrose in 100 mM PB pH 7.4	25	++*	11
184	Hippocampus	100	Copper	100	10% Dextran 10% Sucrose in NMDG pH 7.4	0	X	12
184	Hippocampus	100	Copper	100	10% Dextran 10% Sucrose in NMDG pH 7.4	15	++	13
184	Hippocampus	100	Copper	100	10% Dextran 5% Sucrose 5% Ethylene Glycol in aCSF[a] pH 7.4	20	++	14
144	Cortex	100	Copper	100	20% Dextran in NMDG pH 7.4	25	X	15
144	Cortex	200	Copper	200	10% Dextran 10% Sucrose in NMDG pH 7.4	30	++	16

[$]For vitreous rating, "X", "±", "+" and "++" indicate degree of vitrification. An "X" represents conditions where no areas of the lamella appeared vitreous and tilt series were not able to be collected without stage tracking errors resulting from extreme fluctuations in contrast introduced by ice diffraction. A "±" indicates lamella where tilt series could be acquired, but frames in all tilt series displayed incomplete vitrification evidenced by ice diffraction. A "+" rating was given to conditions where some tilt series could be acquired without ice diffraction, but some non-vitreous ice could be seen in some tilt series. A "++" symbol was assigned to conditions where no or very few (<10%) tilt series displayed evidence of non-vitreous ice.

[+]Dextran 40,000 MW
*Conclusion based on one tilt series
[a]aCSF composition 119 mM NaCl, 26.2 mM $NaHCO_3$, 2.5 mM KCl, 1 mM Na_2HPO_4, 1.3 mM $MgCl_2$, 10 mM glucose, 2.5 mM $CaCl_2$

Table S2. Trench milling parameters for perpendicular and planar cryo-lift-outs from HPF carriers. Related to Star★Methods – Method Details – Cryo-Lift-Out.

Step	Sub-step	Current	Pattern Type	Pattern Dimensions (µm)			Time (min)	Notes
				X	Y	Z		
Fluorescence Overview	NA	NA	NA	NA	NA	NA	20-50	Time for imaging with 1 fluorophore, 20 minutes for 3 Z steps, 50 minutes for 12 Z steps.
Fiducials	NA	4-60 nA	Rectangle	75	4	3	10	Used for alignment between SEM and IFM.
Trenches: Perpendicular	Long Trench	60 nA	RCS	60	150-200	5-6	<30	For a desired lift-out length of ~60-70 um. Longest long trenches should be used in conjunction with deepest deep trenches. For 100 um thick samples, long trench lengths <170 um and deep trench depths <9 should be used to avoid milling into the metal carrier. Largest lift-outs yield ~10-14 3-5 µm thick sections. RCS patterns mill towards target region.
	Deep Trench	60 nA	RCS	60	40-60	6-11		
Trenches: Planar	Side Trenches	200 nA	RCS	50	350-400	6	7–8	For a desired lift-out length of ~350-400 µm. Trenches are milled symmetrically at either side of the region of interest, with the scan pattern directed towards the area for lift out.
	Top Trench	60 nA	RCS	210	50	2	6	To allow the lift-out needle with the copper block attachment to be brought to the top of the sample. The scan pattern is directed towards the area for lift out.
Side and Undercuts: Perpendicular	Side Cuts	15 nA	Rectangle	4	40-60	4	10	Use a stage tilt of <13° to avoid milling into the metal carrier for deep lift-outs.
	Undercut	15 nA	Rectangle	53-58	6-8	4		
Side and Undercuts: Planar	Undercut	15 nA	Rectangle	350-400	6	1	16–20	Perform undercuts from both ±90° stage rotation relative to the region of interest. The time listed is for milling from one of the angles.
Weld Copper Block to Tissue	NA	0.3 nA	CCS	1	2	3	< 5	Tilt the stage to the shallowest angle where remaining material from side cuts is still visible, roughly -3 to 3° . Milling patterns should be oriented to mill from tissue towards the copper block. After attachment, then mill away remaining side material. Dwell time was set to 30 µs
Sectioning and Welding	Sectioning	1-4 nA	Line	~50	NA	4	2-5	The stage was tilted to the shallowest angle possible, around –3 to -5°, for section deposition. 4 nA allows for faster cuts but 1 nA results in a smoother surface that is advantageous for subsequent thinning steps.
	Welding	0.3 nA	CCS	3	0.8	5	3–4 per section	Stage was tiled to 15° before placing welding patterns. Welds were placed with 4 um periodicity such that each section had 3-4 welds per side with 6-8 welds per section in total. CCS patterns milled from the section towards the copper bars. Dwell time was set to 30 µs and each welding pattern took 30 seconds to mill.

Table S3. Datasets generated from vitreous samples collected in this work. Related to Figures 2–7.

Mouse Sex	Mouse Age	Hemisphere (L/R)	Cryoprotectant and Buffer	Incubation Time (min)	Region	Dataset Number (for CA1-sr)	Tilt Series Collected	Synapses	% Dataset that is Synapses	Average Synaptic Vesicle Diameter (nm)
M	184	Not recorded	10% Dextran, 10% Sucrose in NMDG, pH 7.4	15	CA1-sr	1	58	16	28	48.0
M	184	L	10% Dextran, 5% Sucrose, 5% Ethylene Glycol in aCSF, pH 7.4	20	CA1-sr	6	10	3	30	42.2
M	184	L	10% Dextran, 10% Sucrose in aCSF, pH 7.4	20	CA1-sr	5	22	5	23	46.4
F	172	R	10% Dextran, 10% sucrose in NMDG, pH 7.4	20	CA1-sr	2	145	19	13	44.1
M	184	R	10% Dextran, 10% sucrose in NMDG, pH 7.4	15	CA1-sr	3	60	11	18	42.7
F	172	R	10% Dextran, 10% sucrose in NMDG, pH 7.4	20	CA1-sp		35	0	0	N/A
F	172	R	10% Dextran, 10% sucrose in NMDG, pH 7.4	20	CA1-sr	4	64	2	3	45.7
M	184	L	10% Dextran, 10% sucrose in NMDG, pH 7.4	15	CA1*		252	51	20	45.9
F	144	Not recorded	10% Dextran, 10% sucrose in NMDG, pH 7.4	30	cortex		28	0	0	N/A

* CA1 was sampled from CA1-so to CA1-slm
